# Remotely-sensed detection of effects of extreme droughts on gross primary production

**DOI:** 10.1038/srep28269

**Published:** 2016-06-15

**Authors:** Sara Vicca, Manuela Balzarolo, Iolanda Filella, André Granier, Mathias Herbst, Alexander Knohl, Bernard Longdoz, Martina Mund, Zoltan Nagy, Krisztina Pintér, Serge Rambal, Jan Verbesselt, Aleixandre Verger, Achim Zeileis, Chao Zhang, Josep Peñuelas

**Affiliations:** 1 Center of Excellence PLECO (Plant and Vegetation Ecology), Biology Department, University of Antwerp, Universiteitsplein 1, Wilrijk, Belgium; 2 CREAF, Cerdanyola del Vallès 08913, Catalonia, Spain; 3 CSIC, Global Ecology Unit, CREAF-CEAB-UAB, Cerdanyola del Vallés 08913, Catalonia, Spain; 4 UMR 1137 Ecologie et Ecophysiologie Forestières, INRA, 54280 Champenoux, France; 5 Thünen Institute of Climate Smart Agriculture, Bundesallee 50, 38116 Braunschweig, Germany; 6 Bioclimatology, University of Göttingen, Büsgenweg 2, 37077 Göttingen, Germany; 7 Silviculture and Forest Ecology of the Temperate Zones, University of Göttingen, Büsgenweg 1, 37077 Göttingen, Germany; 8 MTA-SZIE Plant Ecology Research Group, Szent István University, Páter K. 1, 2103 Gödöllő, Hungary; 9 CEFE UMR 5175, CNRS - Université de Montpellier - Université Paul-Valéry Montpellier - EPHE, F-34293, Montpellier cedex 5, France; 10 Universidade Federal de Lavras, Departamento de Biologia, CP 3037, CEP 37200-000, Lavras, MG, Brazil; 11 Laboratory of Geo-Information Science and Remote Sensing, Wageningen University, Droevendaalsesteeg 3, Wageningen 6708 PB, The Netherlands; 12 Department of Statistics, Faculty of Economics and Statistics, Universität Innsbruck, Universitätsstr. 15. 6020 Innsbruck, Austria

## Abstract

Severe droughts strongly impact photosynthesis (GPP), and satellite imagery has yet to demonstrate its ability to detect drought effects. Especially changes in vegetation functioning when vegetation state remains unaltered (no browning or defoliation) pose a challenge to satellite-derived indicators. We evaluated the performance of different satellite indicators to detect strong drought effects on GPP in a beech forest in France (Hesse), where vegetation state remained largely unaffected while GPP decreased substantially. We compared the results with three additional sites: a Mediterranean holm oak forest (Puéchabon), a temperate beech forest (Hainich), and a semi-arid grassland (Bugacpuszta). In Hesse, a three-year reduction in GPP following drought was detected only by the Enhanced Vegetation Index (EVI). The Photochemical Reflectance Index (PRI) also detected this drought effect, but only after normalization for absorbed light. In Puéchabon normalized PRI outperformed the other indicators, while the short-term drought effect in Hainich was not detected by any tested indicator. In contrast, most indicators, but not PRI, captured the drought effects in Bugacpuszta. Hence, PRI improved detection of drought effects on GPP in forests and we propose that PRI normalized for absorbed light is considered in future algorithms to estimate GPP from space.

Increased frequency and intensity of drought events is among the prospects that we are facing due to climate change. How ecosystems cope with and respond to extreme droughts will be crucial in the terrestrial feedback to climate change^1^. Severe and recurrent droughts can reduce the terrestrial carbon sink^2^, eliciting a positive feedback on climate change. Ecosystem responses to drought, however, are highly variable in time and space^3^,^4^,^5^. Our knowledge of these responses is still very limited, in part because research on the effects of extreme droughts began only relatively recently and because extreme events occur only rarely in nature. The tools best suited for large-scale, long-term, and continuous high-frequency monitoring of terrestrial ecosystems, i.e. remote sensing imagery, though, have yet to demonstrate their ability to capture the effects of extreme droughts on carbon cycling in natural ecosystems.

To use satellite data for detecting and quantifying the effects of drought at a global scale, we need products that can reliably capture the variation in vegetation state (e.g. defoliation or browning) and in vegetation functioning (e.g. decreases in photosynthesis). Both can change in response to drought, but drought effects may also be restricted to changes in vegetation functioning, for example when trees that already reached peak leaf area experience a drought event, close their stomata and thereby strongly reduce photosynthetic CO_2_ uptake. When drought severity remains below the level where leaves turn brown, vegetation state remains largely unaltered whereas vegetation functioning is altered and gross primary production (GPP) is decreased considerably.

Although commonly used remote sensing indicators such as the Normalized Difference Vegetation Index (NDVI) detect green biomass and generally detect changes in vegetation state (greening, mortality or defoliation)^6^,^7^, these indicators often fail to reflect drought-induced decreases in plant activity. This failure can be due to absence of structural changes, to early saturation at high leaf area index masking moderate structural changes, or to particularly complex structural changes^8^,^9^. A recent comparison of commonly used remote sensing indicators with field observational data accordingly indicated a modest performance of these indicators in capturing the interannual variability in GPP^10^. Whereas for grasslands and evergreen broadleaved forests, NDVI and the Enhanced Vegetation Index (EVI) capture interannual variability in GPP relatively well, the relationships between the relative anomalies in annual GPP (estimated from eddy covariance measurements) and the corresponding anomalies for the remote sensing indicators was very poor for deciduous broadleaved and evergreen needleleaved forests^10^.

The GPP derived from Moderate Resolution Imaging Spectroradiometer (MODIS) product (modGPP) is an advanced satellite product for estimating the uptake of carbon by plants. The modGPP has been compared to field estimates of GPP in various studies, which typically suggest that this product is reliable at large spatial and temporal scales, but is more erratic at fine temporal and spatial resolution^10^,^11^,^12^,^13^. The modGPP is calculated as a function of FAPAR (fraction of absorbed photosynthetically active radiation), temperature, vapor pressure deficit (VPD), light (all three derived from a global dataset^14^) and an estimate of maximum light use efficiency (LUE). It thus includes important abiotic variables that enable short-term fluctuations in this indicator that are not detected in commonly used remote sensing indicators such as FAPAR. Field conditions, however, may deviate from those suggested by these abiotic variables, which may lead to substantial differences between modGPP and field-based GPP estimates during drought periods (see e.g.^11^). The VPD obtained from the global dataset in particular may not well represent water availability. For example, a drought-induced decline in GPP may last much longer than the increase in VPD, which could lead to an underestimation of the drought effect by modGPP if FAPAR is unaltered. Moreover, LUE is here expressed as a biome-specific constant at its potential maximum that may not be representative of the seasonally variable LUE of an ecosystem. Hence, resulting GPP estimates often do not scale with field observations^15^.

It has been suggested that LUE can be estimated remotely from the Photochemical Reflectance Index (PRI)^16^. The PRI is linked to the de-epoxidation status of the xanthophyll cycle pigments, which is one of the components of the non-photochemical de-excitation pathway^16^. PRI can capture the temporal dynamics in LUE via variations in the xanthophyll cycle pigments and the relative ratio of carotenoids to chlorophyll. It is a promising tool to better represent plant functioning through spectral measurements^15^,^17^,^18^. However, a valid remotely-based LUE estimate across different plant functional types and wide range of conditions has not yet been identified^15^,^19^. Accordingly, the performance of PRI in detecting drought effects on plant activity is still debated.

A study by Peguero-Pina *et al*.^20^ demonstrated that ground-based PRI can be a reliable index for detecting drought-induced reductions in plant activity (or more specifically, plant stress indicators such as chlorophyll fluorescence), but drought detection using PRI derived from satellite data may be more problematic^19^. Although PRI can capture changes in the xanthophyll cycle, indicative of stress^21^,^22^, these changes can be obscured by the much stronger signals of seasonal variation in the pigment pool of the leaves (e.g. chlorophyls)^21^, and by seasonal changes in illumination^23^. Recent insights from ground-based remote sensing suggest that a normalization for absorbed light may overcome many of these problems^24^. This promising approach of normalizing PRI for absorbed light remains to be evaluated for satellite-based PRI.

The aim of this study was to test if commonly used remote sensing indicators and PRI calculated with MODIS bands could detect effects of extreme drought on GPP. We hypothesized that commonly used remote sensing indicators, except for modGPP, detect drought effects only when vegetation state is affected. We expected PRI to capture drought effects on GPP in case of physiological changes and in particular, we anticipated a substantial improvement of drought detection by PRI when normalized for absorbed light. To test our hypotheses, we selected a beech forest in Hesse, France where long-term monitoring revealed a strong reduction of GPP during the European heatwave^25^ that was associated with a drought event that lasted from 2003 until 2005 in this forest, but hardly affected vegetation state (leaf area index, tree mortality). To further test the generalizability of our findings, this ideal test case was further complemented with three long-term monitoring sites that had experienced a severe drought. These three additional sites – which were the only ones that met the necessary criteria (see Methods) - are an uneven-aged and unmanaged beech forest in Hainich, Germany; an evergreen broadleaved holm oak forest in Puéchabon, France; and a semi-arid grassland in Bugacpuszta, Hungary (see Supplementary Table S1 for general site information). We used the following remote sensing indicators in our study: NDVI, EVI, EVI2, FAPAR, absorbed photosynthetically active radiation (APAR), Leaf Area Index (LAI), Simple Ratio (SR), Global Environmental Monitoring Index (GEMI), Normalized Difference Water Index (NDWI), modGPP and PRI (see Methods and Supplementary Table S2 for calculations).

## Results

**Figure 1:**
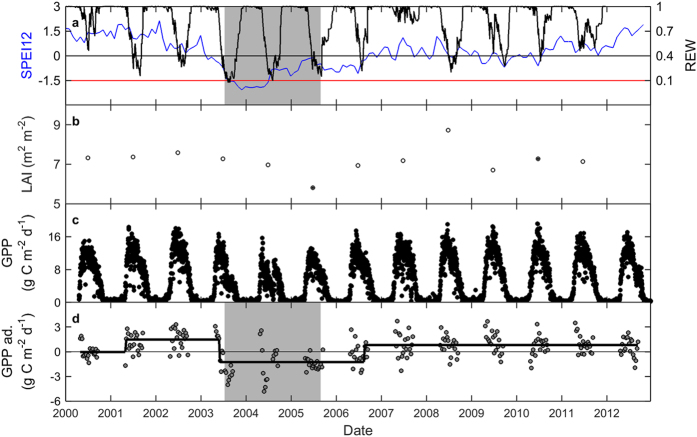
Time series of ground data and of the SPEI12 for the beech forest in Hesse. (**a**) SPEI12 and relative extractable water (REW); (**b**) leaf area index (LAI; determined from trapped litter; see^29^); (**c**) daily values for tower GPP; and (**d**) the results of the breakpoint analysis for GPP (with 8-day values). In (**a**), the blue line corresponds to SPEI12 and the black line to REW. The red line indicates the SPEI12 threshold of −1.5 (indicative of severe drought). Black symbols for LAI indicate the year after thinning. No thinning occurred in the years with open symbols for LAI. In (**d**), the seasonally adjusted data (abbreviated as ‘ad.’ in axis labels) are shown and the black line indicates the results of the breakpoint analysis. The grey area indicates the drought episode that is focused on in this study.

In Hesse, the 2003 heatwave is clearly visible in the Standardized Precipitation Evapotranspiration Index (accumulated over 12 months; SPEI12; see Methods); SPEI12 decreased below −1.5 and did not return to zero until 2006 (Fig. 1a; for temperature and precipitation data, see Supplementary Fig. S1). This corresponds well with patterns for relative extractable soil water (REW; calculated from ground data of soil moisture) which reached a minimum in 2003 and remained low in 2004 and 2005. No other droughts were visible in SPEI12 or REW for the rest of the observation period (Fig. 1a). The strong and lengthy drought had little effect on ground-based leaf area index (LAI) estimates, which varied between 5.8 and 8.7 and reached a minimum in 2005, after the thinning of autumn 2004 (Fig. 1b). In contrast to LAI, tower GPP was substantially reduced during 2003–2005 (Tables 1 and 2) and returned to pre-2003 levels only in 2006 (Fig. 1c). The strong reduction in tower GPP due to the 2003–2005 drought was clearly captured by the test for structural change (Table 1) and by the breakpoint-detection technique (Fig. 1d).

**Figure 2:**
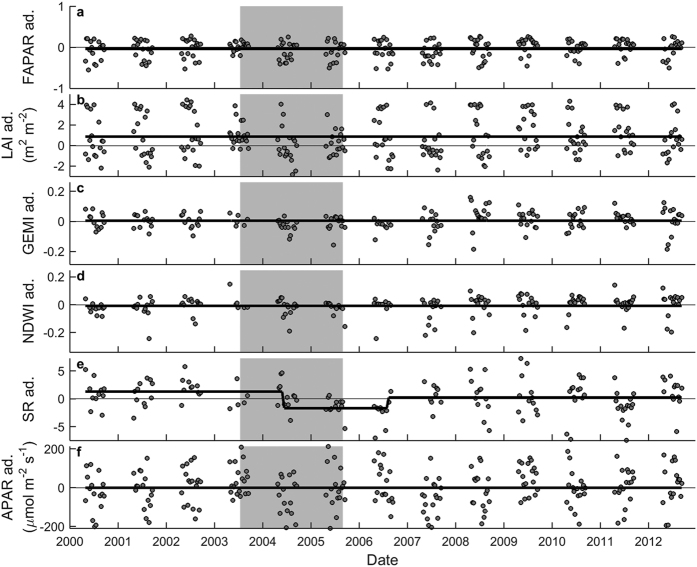
Results of the breakpoint analysis for Hesse. Seasonally adjusted data (abbreviated as ‘ad.’ in axis labels) are shown for the 8-day products of (**a**) MODIS GPP product (modGPP), (**b**) NDVI, (**c**) EVI, (**d**) EVI2, and (**e**) standardized Photochemical Reflectance Index (sPRI). For sPRI normalized for APAR (sPRIn; panel f), the absolute values are shown because no seasonal pattern was detected by sPRIn (see SI). The grey area indicates the drought episode. The black lines indicate the results of the breakpoint analysis.

**Figure 3:**
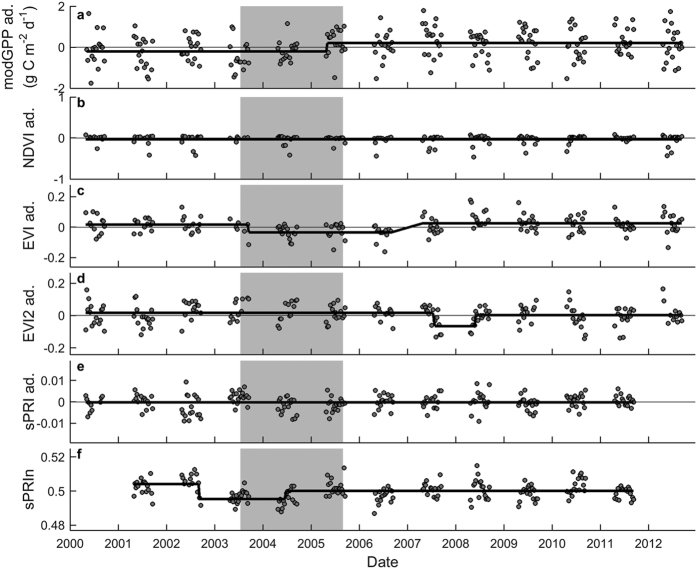
Results of the breakpoint analysis for Hesse. Seasonally adjusted data (abbreviated as ‘ad.’ in axis labels) are shown for the 8-day products of (**a**) FAPAR, (**b**) LAI, (**c**) GEMI, (**d**) NDWI, (**e**) SR, and (**f**) APAR. The grey area indicates the drought episode. The black line indicates the results of the breakpoint analysis.

The GPP product of MODIS did not reproduce the pattern observed for tower GPP. Although the test for structural change indicated a significant difference between drought and non-drought periods (Tables 1 and 2) and a minor break was detected at the end of the drought period, modGPP could obviously not reproduce the field observations; modGPP during drought was even higher than pre-drought modGPP (Fig. 2a, Table 2). Another commonly used reflectance index, NDVI, did not change throughout the time series (Fig. 2b, Table 1). EVI, in contrast, decreased significantly during the drought, as did the simple ratio (SR; for calculation see Table S2), albeit only for 2004–2006 (Fig. 3e, Tables 1 and 2). None of the other commonly used remote sensing indicators decreased during the 2003–2005 drought episode (Figs 2 and 3). We suspected an important role for the blue band in the successful detection of the drought effect with EVI, so we tested if EVI2 (which does not cover the dynamics in the blue band) could detect the drought effect on tower GPP. EVI2 did not decrease during the 2003–2005 drought (Fig. 2d, Tables 1 and 2), supporting the postulated importance of the blue band in EVI.

We further tested if and how the drought effect was reflected in standardized PRI (sPRI; see Eq. 1) and in sPRI normalized for absorbed light (sPRIn; see Eqs 2 and 3). Whereas sPRI showed no significant response to drought, sPRIn decreased significantly in 2003 and 2004 (Fig. 2e,f, Tables 1 and 2). In 2005, i.e., prior to GPP recovery, sPRIn returned to a level similar to pre-2003.

**Figure 4:**
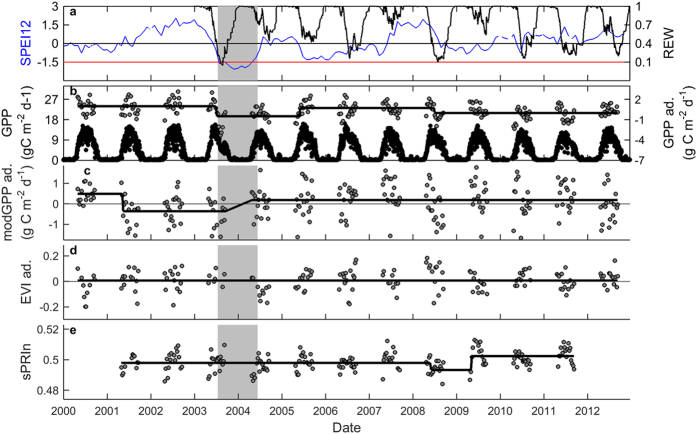
Time series of tower GPP, of remote sensing indicators, and of SPEI12 for the beech forest in Hainich. (**a**) SPEI12 (blue line) and relative extractable water (REW, black line). The red line indicates the SPEI12 threshold of −1.5 (indicative of severe drought); (**b**) daily values of tower GPP, and the results of the breakpoint analysis for 8-day GPP and 8-day products of (**c**) MODIS GPP product (modPP), (**d**) EVI, and (**e**) sPRIn. For tower GPP, black symbols represent the flux measurements, while grey symbols are the data for May-September that were adjusted for the seasonal pattern (abbreviated as ‘ad.’ in axis labels). This adjustment was also made for modGPP and EVI. The grey area indicates the drought episode. The black lines in b-f indicate the result of the breakpoint analysis.

We also tested the performance of the remote sensing indicators at the three other sites where drought had decreased GPP. The first site, Hainich, is an unmanaged beech forest in Germany. Similar to Hesse, SPEI12, REW and GPP in Hainich decreased during the 2003 drought, but this effect was much less pronounced, and soil water was obviously replenished in Hainich in 2004, when REW never decreased below the common stress threshold of 0.4 (Fig. 4). Whereas the reduction in tower GPP at the end of the 2003 growing season can be considered drought-induced, the slightly reduced tower GPP in 2004 as compared to other years most likely resulted from an extraordinary cold and rainy spring and massive fruit production (masting) that was associated with the displacement of leaf buds and thus a lower foliar production in 2004 (Supplementary Table S3).

Similar to Hesse, the modGPP data for Hainich did not match the observations in the field well (Fig. 4). The reason for the lower modGPP in 2001–2003 compared to other years is unclear, but we presume it was related to artifacts in the algorithm, because neither FAPAR (included in the modGPP algorithm) nor any of the other remote sensing indicators showed this pattern. Interestingly, the 2003 drought was not captured by any of the remote sensing indicators (see also Fig. S5). Also, sPRIn did not detect the reduction in photosynthetic activity (Fig. 4e; Tables 1 and 2). On the other hand, the reduction in GPP (associated with a reduction in plant area index; Supplementary Table S3) was somewhat reflected in EVI (Table 1; but detection was not robust across methods and only the test for structural changes comparing 2002–2004 against the rest of the time series revealed an effect) and in GEMI (Global Environmental Monitoring Index; detected in the breakpoint analysis; Table 1 and Supplementary Fig. S5).

**Figure 5:**
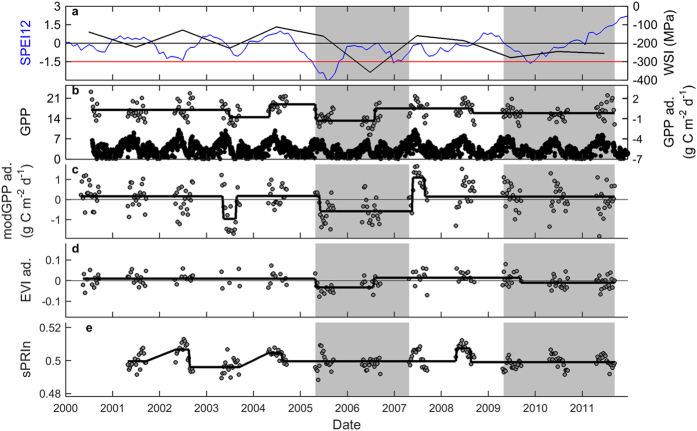
Time series of tower GPP, of remote sensing indicators, and of SPEI12 for the holm oak forest in Puéchabon. (**a**) SPEI12 (blue line) and annual values of the water stress integral (WSI in MPa day, black line). The red line indicates the SPEI12 threshold of −1.5 (indicative of severe drought); (**b**) daily values of tower GPP, and the results of the breakpoint analysis for 8-day GPP and 8-day products of (**c**) MODIS GPP product (modPP), (**d**) EVI, and (**e**) sPRIn. For tower GPP, black symbols represent the flux measurements, while grey symbols are the data for May-September that were adjusted for the seasonal pattern (abbreviated as ‘ad.’ in axis labels). This adjustment was also made for modGPP and EVI. The grey area indicates the drought episode. The black lines in b-f indicate the result of the breakpoint analysis.

In contrast to Hesse, the Mediterranean forest in Puéchabon is exposed to a dry period every year (see REW in Supplementary Fig. S2), causing a decrease in tower GPP every summer, followed by an increase in autumn (Fig. 5b). This typical seasonality is not reflected in SPEI12, which indicates the anomalies of the historical mean season. In 2005, however, SPEI12 decreased below −1.5, indicating a more extreme drought than usual. SPEI12 further suggests a milder drought in 2009. In contrast to Hesse, the 2003 heatwave was not obvious in SPEI12 for Puéchabon. This pattern in SPEI12 corresponded somewhat to the field measurements of predawn leaf water potential, detected by the water stress index (WSI), but contained some important differences. First, according to the WSI, the severest drought occurred in 2006, not 2005 (Fig. 5a). Second, in contrast to SPEI12, WSI did not quickly recover after the drought in 2009 but remained low until 2011. The substantial decrease in WSI in 2005–2006 and the less severe decrease in 2009–2011 were associated with lower GPP (Fig. 5, Table 1). WSI was not extremely low in 2003, but GPP decreased substantially (Fig. 5b) due to the extremely high temperatures (Supplementary Fig. S1). Most commonly used remote-sensing indicators captured the reduction in 2005–2006, but the reduction in GPP associated with the dry period of 2009–2011 was captured only by EVI (Fig. 5d, Table 1). sPRIn decreased for each drought where tower GPP decreased (and also during the 2003 heatwave). sPRIn therefore outcompeted all other indicators.

**Figure 6:**
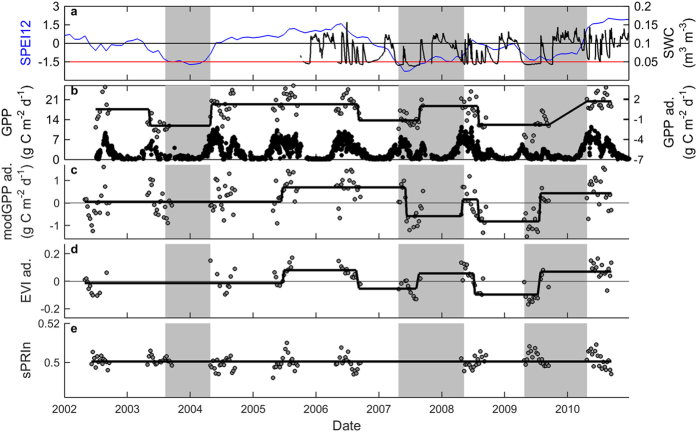
Time series of tower GPP, of remote sensing indicators, and of SPEI12 for the semi-arid grassland in Bugacpuszta. (**a**) SPEI12 (blue line) and soil water content (SWC, black line). The red line indicates the SPEI12 threshold of −1.5 (indicative of severe drought); (**b**) daily values of tower GPP, and the results of the breakpoint analysis for 8-day GPP and 8-day products of (**c**) MODIS GPP product (modGPP), (**d**) EVI, and (**e**) sPRIn. For tower GPP, black symbols represent the flux measurements, while grey symbols are the data for May-September that were adjusted for the seasonal pattern (abbreviated as ‘ad.’ in axis labels). This adjustment was also made for modGPP and EVI. The grey area indicates the drought episode. The black lines in b-f indicate the result of the breakpoint analysis.

Last, we tested drought detection in Bugacpuszta, a semi-arid grassland in Hungary. Similar to Puéchabon, this grassland experiences drought stress every summer, and GPP consequently peaks first in spring and then in late summer, interspersed by a reduction in the dry summer months (Fig. 6). Bugacpuszta experienced extremely dry summers in 2003, 2007, and 2009, as indicated by SPEI12 and supported also by soil moisture measurements. GPP decreased significantly during each of these droughts (Fig. 6b, Tables 1 and 2). The modGPP captured the droughts in 2007 and 2009 but not the 2003 drought. Other commonly used remote sensing indicators such as NDVI and EVI also detected the drought effects of 2007 and 2009 (Table 1 and Supplementary Fig. S5). Interestingly, sPRIn did not show any robust changes throughout the time series (Tables 1 and 2).

## Discussion

The European heatwave of 2003 substantially reduced water availability in Hesse, and also 2004 and 2005 were dry. Despite the lack of a drought response in LAI, tower GPP obviously decreased. Foliar physiology was thus affected by the drought, but massive defoliation or mortality did not occur. The breakpoint detection technique further revealed that the drought effect on tower GPP lasted until end of 2006, even though water levels had returned to pre-drought levels in that year. This illustrates the value of the breakpoint detection technique, which does not require a predefined drought period and is particularly useful to detect and characterize drought impacts and climate-related dynamics^26^. We suggest that the application of this method (previously applied to remote sensing data; e.g. in^27^) to time series of flux data can provide deeper insight into ecosystem responses to drought.

As expected, most commonly used indicators did not change throughout the time series. The lack of response of NDVI, FAPAR, and other of these commonly used indicators corresponded to the lack of response of LAI. Moreover, the signals are quickly saturated in forests with high LAI (such as Hesse), making these indicators insensitive to small changes in foliar biomass. Surprisingly, however, modGPP did not decrease in response to the severe drought. Given that the algorithm for this product includes temperature and VPD^28^, and both increased in Hesse during the 2003 heatwave^29^, modGPP was expected to detect at least a small decrease in 2003. These results indicate that modGPP cannot reliably estimate the tower GPP response to drought for the beech forest in Hesse.

Of the commonly used remote sensing indicators tested, only EVI and simple ratio (SR) decreased significantly during the drought period. Both indicators are less prone to saturation problems^30^. Because the reduction in SR lagged behind the reduction in tower GPP and was not noticeable before 2005, we presume that the reduction in SR mainly detected the thinning effect on LAI and not the drought response of tower GPP. EVI, on the other hand, had already decreased in 2004, and did not even respond to the thinning event of 2005. EVI thus captured the drought-induced reduction in GPP in the absence of a change in LAI.

EVI was the only commonly used indicator under test that includes the blue band, which was included in EVI to reduce the effect of atmospheric aerosols but could also provide additional information on foliar carotenoid contents. Carotenoid absorption peaks between 400 and 500 nm (covered by the blue band)^31^, and the carotenoid:chlorophyll ratio usually increases in response to environmental stresses^32^, which results in a decrease in EVI. The fact that EVI2 (which is related to EVI but does not include blue band reflectance) remained unaffected by the 2003–2005 drought episode, supports our postulation that the reduction in EVI was related to an increase in foliar carotenoid:chlorophyll ratio that was captured by the blue band.

sPRI did not change throughout the time series for Hesse. When normalized for absorbed light, however, sPRIn did show a decrease in 2003 and this reduction persisted during 2004. The importance of the normalization for detecting the drought effect can be explained as follows: sPRI can capture changes in the xanthophyll cycle (which responds quickly to environmental stresses), but these changes are obscured by the much stronger signals of seasonal variation in the whole pool of pigments in the leaves^21^, and by seasonal changes in illumination^23^. Normalization is therefore necessary to remove the signal of seasonality in pigment pool and foliar structure to clarify the signal of the xanthophyll cycle^24^. PRI was normalized for absorbed light to remove (most of) the signal caused by changes in the pigment pool and illumination, and the resulting sPRIn should thus reflect changes in the xanthophyll cycle. Water limitation was somewhat alleviated in 2005, and sPRIn returned to pre-2003 levels. In contrast to EVI and SR, sPRIn seemed thus unaffected by the thinning in 2005, further confirming its performance in representing the functioning, but not the state, of the vegetation.

Hainich, a mixed beech forest in Germany, also experienced a drought in 2003, but unlike the case for Hesse, this event was brief; soil water was fully replenished during the winter of 2003–2004. Leaf physiology was probably affected only briefly, which poses a challenge on drought detection from satellite data. Indeed, the drought-induced reduction in tower GPP in autumn 2003 was not detected by any of the remote-sensing indicators evaluated (we did not consider modGPP, which obviously did not well reproduce the field observations for previous years) and this example thus indicates the limits of drought detection from satellite imagery. The timing of the drought at the end of the season may have complicated the detection of its effects, with relatively few data points available during the brief drought, and the regular leaf senescence may have obscured drought detection even further.

Interestingly, a reduction in GPP in 2004, related most likely to reduced foliar biomass production caused by an extraordinarily cold and rainy spring (Supplementary Fig. S1) and masting in 2004^33^, was detected to some extent by EVI and especially by GEMI (Supplementary Fig. S5), but not by sPRIn. This illustrates the added value of sPRIn, as EVI and GEMI alone could be taken to suggest a carry-over effect of the drought, whereas the lack of a change in sPRIn indicates that this change in GPP was due to a structural change, not to a change in leaf functioning.

The evergreen broadleaved forest in Puéchabon experienced recurrent droughts during the study period. GPP decreased most strongly during the 2005–2006 drought, which was clearly detected by most remote-sensing indicators. Neither LAI, nor foliar production decreased in 2006^34^, whereas decreases in GPP are directly controlled by water limitation^35^. On the other hand, sPRIn was the only indicator that detected the less pronounced drought in 2009–2011 and the reduction of tower GPP during the 2003 heatwave. The inability of modGPP to detect the drought effects on tower GPP in 2009–2011 may be related to the meteorological data, which in our case differed from field observations.

Lastly, GPP in the Hungarian grassland decreased during each of the three drought events, and these decreases were well detected in many commonly used remote sensing indicators, but not in sPRIn. The contrasting performance of different remote sensing indicators between the grassland and forests studied here could be related to the earlier observations that grasslands respond differently to drought than forests. Whereas trees close their stomata and thus reduce tower GPP when soil moisture decreases below the comfortable level, thereby maintaining leaf water potential above a critical threshold and avoiding cavitation, grasses are not conservative in their water use and tend to remain active until moisture levels drop below the wilting point^36^. Grasses subsequently turn brown and die. Grasslands may thus respond to drought in a way that is more easily detected by remote sensing indicators such as NDVI that reflect green biomass. The quick transition of grasslands from active to dead vegetation may complicate detection by eight-day PRI indicators. The performance of PRI has also been suggested to depend highly on the density of the vegetation^37^. View angle had very little effect on PRI for dense forests, but may confound PRI for sparser vegetation due to the increased importance of background reflectance^37^.

In summary, evaluation of sPRIn and commonly used remote sensing indicators against four sites where a severe drought event reduced GPP revealed (1) that commonly used remote sensing indicators, including modGPP are likely to leave drought effects on GPP in forests undetected, (2) that sPRIn is a promising indicator that allows drought detection from space and provides useful information about vegetation functioning that is not captured by the other remote sensing indicators under test, and (3) that GPP responses in a seasonally dry grassland are best detected by greenness indicators such as NDVI, whereas the utility of sPRIn is limited. The fact that drought effects on GPP were in several cases not detected by modGPP contrasts somewhat with Reichstein *et al*.^38^, who found a strong reduction in modGPP across Europe during the 2003 heatwave. This apparent discrepancy can be clarified by the difference in spatial resolution and scale. While Reichstein *et al*.^38^ reported changes across Europe, using 10 × 10 km pixel resolution, we zoom in on just a few 1 × 1 km pixels in Europe. Given that for example the forest in Hesse is relatively small and surrounded by cropland and grassland, the difference between our studies highlights the importance of choice of resolution: use of small pixels is essential for a more mechanistic understanding, whereas large pixels facilitate estimation of large-scale impacts, although following our study, the latter might underestimate the drought impact on GPP.

A global product for estimating GPP from space would obviously be of great value for understanding and predicting terrestrial carbon cycling. However, the most advanced product currently available - modGPP - can not explain substantial spatial and temporal variation in GPP^10^ and often fails to accurately capture pronounced drought effects on GPP^39^. This illustrates the broader problem that modGPP (and other commonly used remote sensing indicators) reflect primarily changes in vegetation state and leave important changes in vegetation functioning undetected. The drought response of modGPP is modeled on meteorological data (temperature and VPD), and these may not represent the drought as experienced by the biota, which depends not only on incoming water, but also on soil characteristics and rooting depth^40^. Obviously, if modGPP cannot capture strong drought effects, it is unlikely to capture even more subtle changes with possible important implications for the terrestrial carbon cycle. Remote sensing products targeting drought responses but based solely on greenness indicators and meteorological data (e.g. the Vegetation Drought Response Index^41^) may be prone to similar problems as modGPP. We suggest that remote sensing products to estimate GPP be based on a combination of indicators of vegetation state (e.g. FAPAR) and functioning (e.g. sPRIn).

The PRI has been successfully incorporated in remote sensing algorithms to estimate GPP of a specific site from space^42^, and this GPP estimate was more accurate than modGPP for the Mediterranean forest under test (Castelporziano, Italy). Until now, however, a key limitation of PRI is that its relationship with plant physiological variables such as LUE varies spatially and temporally^43^,^44^, complicating its incorporation in remote sensing algorithms for wider application. Possibly, sPRIn has more robust spatiotemporal patterns because at least part of the complicating factors typical for PRI are excluded through the normalization that removes the influence of variation in pigments and illumination. A thorough evaluation of the overall spatiotemporal performance of sPRIn should enable further improvement of the algorithms for estimating GPP from space and future studies need to evaluate such product against modGPP and alternatives as e.g. provided by^39^.

Direct assessment of photosynthetic activity and LUE with PRI remains thus an area of ongoing research, requiring a careful consideration of the spatial and temporal factors affecting the PRI components, and a thorough evaluation of sPRIn. Different ecosystems having contrasting constraints on carbon fluxes should also have contrasting optical properties reflected in PRI and NDVI dynamics^45^. It is not likely that any single parameterization will apply equally well across all ecosystems, biomes and/or seasons due to the contrasting controls on physiology and structure, but this is an open question that needs further consideration in the immediate future.

## Methods

### Site description

The key site in this study was a beech forest in Hesse, northeastern France. The monitoring site was installed in a 0.6-ha fenced experimental plot in the central part of the forest. The forest was naturally regenerated and contained 95% European beech (*Fagus sylvatica* L.) trees averaging 40 years of age in 2005. The young forest has been managed traditionally by the French forest service (ONF) and was thinned just before the start of the monitoring (end of 1995, no data available), in spring 1999, at the end of 2004, and at the beginning of 2010. Stem density was reduced by 24.4, 17.7 and 10.5%, whereas LAI decreased by 35.1, 33.3 and 22.9%, in 1999, 2004 and 2010, respectively (Fig. 1b). More information about this forest is provided in Supplementary Table S1 and in Bréda *et al*.^46^ and Granier *et al*.^29^.

We further searched the FLUXNET and European fluxes databases for sites to test the robustness of the results obtained for Hesse. These sites, as for Hesse, had to meet the following criteria:

1. Time series of GPP covering at least five consecutive years
2. Homogenous vegetation at 1 km resolution
3. Drought event that is detected in GPP

The first criterion is a prerequisite for analyzing time series. The effect of a drought on GPP and its detection by remote sensing imagery can only be tested with sufficiently long time series that (1) enable comparison of drought and non-drought periods, and (2) enable detection of potential lagged and/or carry-over effects. The second criterion ensures reliable comparisons between field data and remote sensing indicators, as it avoids contradictory flux measurements that depend on wind direction and footprint extent. The third criterion is necessary for testing the use of satellite data for detecting the effects of drought. We selected only those sites where GPP was significantly affected during a drought based on a breakpoint-detection technique and for which the site investigators confirmed that the reduction in GPP during the drought could indeed be attributed to water stress. These three criteria excluded the vast majority of sites, especially because drought events were often not reflected in GPP measurements (e.g. because trees had access to deep soil water), or because simultaneous management activities confounded the drought response of GPP. The remaining sites provided datasets of high quality, which was essential for this study. These three additional sites were: an uneven-aged and unmanaged beech forest in Hainich, Germany; an evergreen broadleaved holm oak forest in Puéchabon, France; and a semi-arid grassland in Bugacpuszta, Hungary.

### Drought episodes

We calculated the Standardized Precipitation Evapotranspiration Index (SPEI) using the SPEI-package in R^47^. SPEI is a meteorological drought indicator based on a monthly climatic water balance (precipitation – potential evapotranspiration), which is adjusted using a three-parameter log-logistic distribution. We then accumulated the values at a timescale of 12 months, and this SPEI12 indicates for each moment in time the meteorological dryness (or wetness) of the previous 12 months as compared to historical observations. The choice of this timescale was arbitrary but of minor importance for this study, because it only served to identify the occurrence and timing of a severe drought. Moreover, the patterns in SPEI were similar for all timescales between six and 18 months (data not shown). SPEI values between 1 and −1 are considered normal, whereas values <−1 indicate drought and values <−1.5 indicate severe drought^47^. We defined a drought as a period where SPEI12 decreased to ≤−1.5.

Because we were interested in evaluating the use of globally available products, we extracted the necessary long-term precipitation and potential evapotranspiration data from EC-JRC-MARS (a dataset based on ECMWF model outputs and a reanalysis of ERA-Interim; see http://spirits.jrc.ec.europa.eu/), based on the geographic location of each site. This dataset provides a finer spatial resolution (0.25°) than the CRU data set (0.5°) used by Vicente Serrano *et al*.^47^. The period covered by the JRC-MARS dataset was 1989–2012. Potential evapotranspiration estimates in this dataset are calculated using the Penman-Monteith equation^48^.

Although SPEI is a useful and convenient metric for detecting meteorological drought, with good spatial and temporal coverage, it does not necessarily indicate field conditions well. Plants experience a given reduction in precipitation quite differently between sites (e.g., because of differences in soil texture)^40^, and periods of drought are therefore best identified using site-specific data. For that reason, we computed for the selected sites relative extractable water (REW) based on a water balance model^49^ (see details in Supplementary Notes S1). REW represents soil moisture as a fraction of maximum soil water across the rooting zone and is thus a type of agricultural drought index^50^. Below REW = 0.4, plants can be considered drought stressed^29^.

Because the necessary data for calculation of REW were not available for Bugacpuszta, soil water content (SWC) was used instead as an indicator of the water status of the site. These data suggested a strong drought in 2009, when SPEI12 remained only slightly above −1.5. Therefore, 2009 was also considered as a dry year in the analyses. In addition, we computed for Puéchabon the water stress index (WSI), which incorporates not only soil measurements but also predawn leaf water potential measurements and is therefore an even better indicator of plant drought stress than REW. We consider WSI ≤ −250 MPa day to be indicative of moderate to severe drought (see Supplementary Notes S1 for details).

### Flux data

We extracted half-hourly data from the European fluxes database (http://www.europe-fluxdata.eu), and complemented these with the most recent data available (provided by the PI). We then calculated daily means from the half-hourly data. Gaps in the data due to sensor malfunctions or less than ideal turbulence conditions^51^ were filled using marginal distribution sampling described by Reichstein *et al*.^52^. We used only days when both daily meteorological and flux data contained less than 20% gapfilled half-hourly data. Gross primary production was estimated using flux partitioning, based on the extrapolation of nighttime flux observations corrected for temperature differences with temperature-dependency relationships^52^. We further refer to this GPP estimate as ‘tower GPP’ to distinguish from MODIS’ GPP product (modGPP; see below).

### Satellite data

We used MODIS subset data (collection 5) provided by ORNL DAAC (http://daac.ornl.gov/cgi-bin/MODIS/GR_col5_1/mod_viz.html, col. 5) for deriving the commonly used indicators (Table S2). The MOD09A1 ASCII subset dataset contains surface reflectance data of MODIS bands ranging from 620 nm to 2155 nm at 500 m spatial resolution and eight-day synthesis period. For comparison with the other satellite indicators at 1-km spatial sampling, the 500 m reflectance data were re-sampled to 1-km spatial resolution by simple aggregation of the 2 × 2 pixels centered at the flux tower. Only high quality data were included and images affected by clouds or snow cover were removed according to the quality flag associated with MOD09A1 data. The reflectances at 1 km spatial resolution were used to compute the standard MODIS indicators presented in Table S2 (NDVI, EVI, EVI2, SR, GEMI, NDWI). We also gathered FAPAR and LAI from MOD15A2^53^ at 8 days and 1 km and GPP data from MOD17 at 8 days and 500 m, aggregated at 1 km; http://www.ntsg.umt.edu/project/mod17^28^. Finally, APAR (absorbed photosynthetically active radiation) was calculated from the MODIS FAPAR data, and global radiation was calculated from EC-MARS-JRC data (0.25° spatial resolution; one-day interval). We assumed a constant fraction of PAR in global radiation: global radiation was divided by 43 to obtain an estimate of the photosynthetically active radiation (PAR; this relationship was derived from a comparison with field observations of PAR; Fig. S3). This method removed gaps in the field data of PAR and was especially relevant for the current study where the aim was to determine how drought effects observed in the field could be detected using global databases. Note that we did not gapfill MODIS data because the interpolations that are used for gapfilling could create important artefacts that can influence drought detection.

Calculations of PRI were based on MOD21KM daily-calibrated radiance data (see http://ladsweb.nascom.nasa.gov). These images were georeferenced using MOD3 geolocation information and the Swath MODIS Tool available from LP DAAC (https://lpdaac.usgs.gov/tools/modis_reprojection_tool_swath). The pixel area of 1 × 1 km corresponding to the flux tower position was extracted and PRI was calculated as suggested by its developers^16^,^54^ (see Supplementary Table S2) and later also applied by, for example, Drolet *et al*.^17^, Garbulsky *et al*.^42^, Goerner *et al*.^44^, Guarini *et al*.^55^.

Dates when clouds were detected (using the MODIS MOD35 cloud algorithm and visual inspection) were discarded. The maximum value of PRI was calculated from a daily time series over an eight-day window to correspond to the temporal resolution of MOD09 and MOD15A2. Because corrected reflectances for applying an atmospheric correction are currently not available, we used the uncorrected PRI. Earlier studies reported that atmospheric correction did not improve the estimation of LUE^17^,^42^,^44^, and therefore MODIS PRI without atmospheric correction was assumed to be an accurate indicator of LUE in different ecosystem types^17^,^42^,^55^. The temporal consistency observed in the PRI profiles presented in our study supports the reliability of the estimates.

To ensure positive values that are better comparable to commonly used remote sensing indicators such as NDVI, PRI values were standardized as by Rahman *et al*.^56^ and Goerner *et al*.^57^:


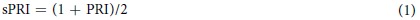


Because the relationship between PRI and LUE varies over the seasons^24^, along with the dynamics in green biomass that absorbs the incoming radiation, we calculated a standardized PRI (PRIn), which is normalized for APAR (absorbed PAR; see^24^ for rationale). This normalization allows to focus on the part of PRI explaining LUE and photosynthetic performance because it removes the part linked to pigments and structure. PRIn was calculated by:


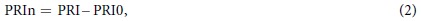


with PRI0 the intercept of PRI vs APAR for a two-month window. This window size was the best compromise between having sufficient data for a reliable fit (eight data points if none are missing), and an informative relationship between PRI and APAR. The latter changes over time^24^, so the smaller the window, the better APAR represents the structure of the vegetation. Finally, we standardized PRIn to obtain only positive values analogous to sPRI:


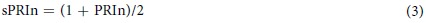


sPRIn is an indicator of the excess energy not photosynthetically processed; when plants are stressed, their LUE decreases as more energy is lost through heat dissipation. An increase in sPRIn should thus indicate an increase in foliar photosynthetic activity.

### Analyses

To test whether drought effects on tower GPP are detected by any of the remote sensing indicators under test, we first calculated for each site the average seasonal pattern over the entire time series, using a 1-month moving window (thus providing for each day the average value across the time series). The anomalies from this average season were then computed and used for further analyses, except in the case of sPRIn, which showed no seasonality (Supplementary Fig. S4). We then removed for all sites data for October-April (thus leaving only May-September) to study only the season when plants have leaves and are active. This elimination was necessary to avoid artifacts in the remote sensing indicators caused by sensing soil instead of leaves.

In order to test if tower GPP and the remote sensing indicators were significantly different during the drought period(s) as compared to the rest of the time series, we compared a linear model with and without a drought term (which indicates the pre-defined drought periods visualized as grey areas in Figs 1–7) using ANOVA. In addition, we ran a test for structural changes^58^ considering only the pre-defined drought period and the year before. As the data were all seasonally adjusted prior to this analysis, a simple piecewise constant model was used. Hence, whereas the ANOVA considers all drought periods within a time series at once, the test for structural changes considers the individual drought periods.

Responses in remote sensing indicators may lag behind the responses observed in the field - for example when physiological changes reduce tower GPP while vegetation state (LAI for example) is unaltered -, and full recovery from drought may be (long) after precipitation ends the meteorological drought. These inconsistent responses complicate robust detection of drought effects and are best analyzed with a flexible technique, such as breakpoint analysis. Breakpoint analysis is a technique developed for detecting breaks based on structural changes in the time series^59^. This analysis is related to the test for structural changes indicated above, but does not presume any periods where breaks would occur (i.e., no pre-defined drought periods are considered). A simple piecewise constant model with unknown number and location of breakpoints was adopted. Given a certain number of breaks, their location is chosen to minimize the residual sum of squares across the corresponding segments subject to a minimal segment size of 12 observations (about three months of observations). The number of breaks was then chosen to optimize the Bayesian information criterion (BIC). The result for each site and anomaly series is a piecewise constant fit with the BIC-optimal number/location of breaks/segments. See Zeileis *et al*.^60^ for more details.

All analyses were performed in MATLAB R2014b (The MathWorks Inc., Natick, USA), except for the breakpoint analysis and the test for structural change, which both used the strucchange package in R^58^.

## Additional Information

**How to cite this article**: Vicca, S. *et al*. Remotely-sensed detection of effects of extreme droughts on gross primary production. *Sci. Rep.* **6**, 28269; doi: 10.1038/srep28269 (2016).

## Supplementary Material

Supplementary Information

## Figures and Tables

**Table 1:** For each site and each variable the P values of ANOVA analysis comparing a model with and without drought term and P values resulting from the test for structural changes (SC) for the indicated period (i.e., the drought episode + the year before).

	Hesse			Hainich			Puéchabon			Bugacpuszta			
	ANOVA	SC (2002–2006)	ANOVA	SC (2002–2003)	SC (2002–2004)	ANOVA	SC (2004–2006)	SC (2008–2011)	ANOVA	SC (2002–2003)	SC (2006–2007)	SC (2008–2009)	
**GPP**	<0.01	<**0.01**	<0.01	<**0.01**	<**0.01**	<0.01	<**0.01**	**0.03**	<0.01	<**0.01**	<**0.01**	<**0.01**
**modGPP**	<0.01	0.05	0.01	0.15	0.37	<0.01	<**0.01**	0.07	<0.01	0.15	<**0.01**	<**0.01**
**EVI**	<0.01	<**0.01**	0.74	0.99	<0.01	<0.01	<**0.01**	<**0.01**	<0.01	NA	<**0.01**	<**0.01**
**sPRIn**	0.03	<**0.01**	0.21	0.12	0.36	<0.01	<**0.01**	<**0.01**	0.10	0.99	NA	0.01
**APAR**	0.90	0.30	0.76	0.44	0.48	0.04	**0.02**	0.52	<0.01	**0.03**	0.07	<**0.01**
**EVI2**	0.08	0.77	0.63	0.21	0.79	0.08	0.90	0.10	0.02	0.74	<**0.01**	**0.02**
**FAPAR**	0.53	0.48	0.52	0.63	0.45	<0.01	0.38	1.00	<0.01	<0.01	<**0.01**	**0.01**
**GEMI**	0.05	0.27	0.22	0.98	**0.01**	0.02	0.15	<0.01	<0.01	NA	<**0.01**	<**0.01**
**LAI**	0.07	<0.01	0.53	0.32	0.10	<0.01	<**0.01**	0.99	<0.01	<0.01	<**0.01**	<**0.01**
**NDVI**	0.90	0.90	0.26	0.78	0.90	<0.01	<**0.01**	0.05	<0.01	NA	<**0.01**	<**0.01**
**NDWI**	0.62	0.06	0.41	0.97	0.97	<0.01	<**0.01**	0.01	<0.01	NA	<**0.01**	<**0.01**
**sPRI**	0.25	0.37	0.19	0.78	0.57	0.25	0.41	0.12	<0.01	0.03	0.69	0.99
**SR**	0.04	<**0.01**	0.94	1.00	0.67	<0.01	<**0.01**	0.04	<0.01	NA	<**0.01**	<**0.01**

P values in bold indicate robust reductions during the drought period (i.e. the structural change test corresponds to the results of the breakpoint analysis presented in Figs 2–7 and in SI). NA indicates that too few data were available to run the test for structural changes.

**Table 2:** For each site and each variable the mean and standard deviation (in brackets) of the anomalies from the average season for the periods without drought (white areas in Figs 1, 2, 3, 4, 5, 6), for the drought periods (grey areas in Figs 1, 2, 3, 4, 5, 6) and for the period preceding the first drought.

	Hesse			Hainich			Puéchabon			Bugacpuszta		
	No drought	Pre-drought	Drought	No drought	Pre-drought	Drought	No drought	Pre-drought	Drought	No drought	Pre-drought	Drought
**GPP**	0.72 (1.31)	0.88 (1.32)	**−1.65 (1.57)**	0.46 (1.04)	0.99 (1)	**−0.74 (1.33)**	0.42 (1.13)	0.36 (1.17)	**−0.48 (1.12)**	0.73 (1.74)	−0.53 (2.17)	**−1.3 (1.41)**
**modGPP**	0.08 (0.72)	−0.18 (0.73)	**−0.07 (0.6)**	0.11 (0.75)	−0.08 (0.69)	**−0.29 (0.7)**	0.17 (0.72)	0.03 (0.69)	**−0.15 (0.68)**	0.27 (0.66)	−0.01 (0.62)	**−0.31 (0.64)**
**EVI**	0.02 (0.06)	0.02 (0.05)	**−0.03 (0.05)**	0.01 (0.08)	0 (0.09)	0.02 (0.04)	0.01 (0.03)	0.01 (0.03)	**−0.01 (0.03)**	0.03 (0.07)	−0.04 (0.06)	**−0.05 (0.08)**
sPRIn*100	50.05 (0.52)	50.14 (0.59)	**49.86 (0.48)**	49.89 (0.56)	49.81 (0.6)	49.64 (0.67)	50.16 (0.55)	50.07 (0.55)	**49.88 (0.38)**	50.02 (0.34)	50.1 (0.23)	50.17 (0.29)
**APAR**	−0.35 (94.32)	−3.98 (91.73)	−4.91 (105.97)	−4.38 (63.88)	−2.04 (71.65)	−2.39 (81.01)	−2.57 (49.04)	−0.77 (48.41)	**1.41 (49.1)**	14.54 (48.49)	−11.25 (42.17)	**−19.28 (46.15)**
**EVI2**	0 (0.06)	0.01 (0.06)	0.03 (0.06)	0.01 (0.06)	0.01 (0.06)	0.02 (0.08)	0.01 (0.06)	0.02 (0.06)	0.01 (0.06)	0.02 (0.06)	0.04 (0.07)	**0.01 (0.08)**
**FAPAR**	−0.03 (0.21)	−0.04 (0.22)	−0.06 (0.21)	−0.02 (0.12)	−0.02 (0.13)	−0.05 (0.15)	−0.01 (0.05)	−0.02 (0.05)	**−0.02 (0.05)**	0.03 (0.1)	−0.03 (0.09)	**−0.08 (0.11)**
**GEMI**	0.01 (0.06)	0.01 (0.05)	−0.02 (0.05)	0 (0.08)	−0.01 (0.08)	−0.09 (0.14)	0.01 (0.04)	0.01 (0.04)	**−0.01 (0.03)**	0.03 (0.06)	−0.03 (0.05)	**−0.04 (0.07)**
**LAI**	0.98 (1.91)	1.03 (2)	**0.29 (1.45)**	−0.23 (1.08)	−0.14 (1.22)	−0.51 (1.38)	−0.01 (1.06)	−0.08 (1.01)	**−0.24 (1.03)**	0.1 (0.32)	−0.09 (0.27)	**−0.21 (0.26)**
**NDVI**	−0.04 (0.12)	−0.03 (0.12)	−0.03 (0.1)	−0.03 (0.15)	−0.03 (0.17)	−0.2 (0.28)	0.01 (0.03)	0.01 (0.03)	**−0.01 (0.03)**	0.05 (0.1)	−0.04 (0.09)	**−0.09 (0.12)**
**NDWI**	−0.01 (0.07)	−0.01 (0.06)	−0.02 (0.05)	−0.02 (0.1)	−0.02 (0.11)	−0.11 (0.18)	0.01 (0.05)	0 (0.05)	**−0.01 (0.04)**	0.05 (0.14)	−0.12 (0.11)	**−0.1 (0.13)**
sPRI*100	0 (0.35)	−0.05 (0.42)	−0.08 (0.36)	−0.01 (0.37)	−0.08 (0.43)	−0.07 (0.31)	−0.03 (0.27)	−0.04 (0.29)	−0.02 (0.23)	−0.06 (0.26)	−0.06 (0.29)	**0.14 (0.23)**
**SR**	0.27 (2.9)	1.2 (2.19)	**−0.15 (1.97)**	−0.63 (7.52)	−0.58 (8.34)	−3.2 (8.2)	0.16 (0.55)	0.15 (0.63)	**−0.17 (0.55)**	0.54 (1.21)	−0.35 (0.94)	**−0.71 (1.14)**

For sPRIn for which the absolute values are presented. For sPRI and sPRIn, values are multiplied by 100 for visualization purposes. Bold numbers indicate significant differences between drought and non-drought periods (corresponding p values are in Table 1, in columns with header ‘ANOVA’).
