# Supplementary material for: Remotely-sensed detection of effects of extreme droughts on gross primary production
Source: Sci Rep. 2016 Jun 15;6:28269. doi: 10.1038/srep28269 (PMC4908591; doi:10.1038/srep28269)
Supplement: Supplementary Information [file srep28269-s1.pdf]

Supplementary Information

Article title: Remotely-sensed detection of effects of extreme droughts on gross primary production

Authors: Sara Vicca, Manuela Balzarolo, Iolanda Filella, André Granier, Mathias Herbst, Alexander Knohl, Bernard Longdoz, Martina Mund, Zoltan Nagy, Krisztina Pintér, Serge Rambal, Jan Verbesselt, Aleixandre Verger, Achim Zeileis, Chao Zhang, Josep Peñuelas

The following Supporting Information is available for this article:

Table S1 Main characteristics of the study sites

Table S2 Description of the formulae used to derive the remote sensing indicators used in the study.

Table S3 Field data for Hainich indicative of plant and fruit production.

Fig. S1 Time series of monthly precipitation and mean monthly air temperature for the four sites under study.

Fig. S2 Relative extractable water (REW) for Puéchabon (black line). The red line indicates the common threshold of 0.4, indicative of drought stress. See manuscript text for further details.

Fig. S3 Relationship between photosynthetically active radiation (PAR; obtained from flux tower) and global radiation (Rg; from JRC-MARS).

Fig. S4 Seasonal patterns for the time series of GPP and of the different remote sensing products tested in this study. Black symbols indicate the measurements, while the red line represents an average season (using a ~10-day moving window) that was used to calculate the anomalies of the seasonal pattern (further used in the bfast analysis, except for sPRIn, which had no clear seasonality).

Fig. S5 Breakpoint analysis for remote sensing products that were not shown in the manuscript. SPEI is also shown.

Notes S1 Water availability metrics

Table S1: Main characteristics of the study sites: location, elevation (El), mean annual temperature (MAT), mean annual precipitation (MAP), vegetation type and dominant species and a key reference.

Site	Country	Location	El (m)	MAT (°C)	MAP (mm)	Vegetation (species)	Reference
Hesse	France	48.6742 N, 7.0656 E	300	9.2	885	Deciduous broadleaved forest (*Fagus sylvatica*)	[1]
Puéchabon	France	43.7414 N, 3.5958 E	61	13.4	883	Evergreen broadleaved forest (*Quercus ilex*)	[2]
Bugacpuszta	Hungary	46.6911 N, 19.6013 E	106	10.4	562	Semi-arid grassland (*Festuca pseudovina*, *Carex stenophylla*, *Salvia pratensis*)	[3]
Hainich	Germany	51.0793 N, 10.45 1E	445	7	800	Deciduous broadleaved forest (*Fagus sylvatica*)	[4]

Table S2: Description of the formulae used to derive the remote sensing indicators. The variable ‘b’ represents the reflectance values derived from MODIS: b1: 620 – 670 nm; b2: 841 – 876 nm; b3: 459 – 479 nm; b7: 2105 – 2155 nm, b11: 526 – 536 nm; b12: 546 – 556 nm.

Index	Formula	Use	Reference
Normalized Difference Vegetation Index (NDVI)	$\frac{b2-b1}{b2+b2}$	Greenness / biomass	[5]
Enhanced Vegetation Index (EVI)	$2.5\cdot\frac{(b2-b1)}{b2+6\cdot b1-7.5\cdot b3+1}$	Greenness / biomass	[5]
Enhanced Vegetation Index 2 (EVI2)	$2.5\cdot\frac{(b2-b1)}{b2+2.4\cdot b1+1}$	Greenness / biomass	[6]
Simple Ratio (SR)	$\frac{b2}{b1}$	Greenness / biomass	[5]
Global Environmental Monitoring Index (GEMI)	$\eta(1-0.25\eta)-\frac{b1-0.125}{1-b1}$ $\eta=\frac{2(b2^2-b1^2)+1.5\cdot b1+0.5b1}{b2+b1+0.5}$	Greenness / biomass	[7]
Normalized Difference Water Index (NDWI)	$\frac{b2-b7}{b2+b7}$	Leaf / canopy water content	[8]
Photochemical Reflectance Index (PRI)	$\frac{b11-b12}{b11+b12}$	Changes in carotenoid pigments (e.g. xanthophyll pigments)	[9]

Table S3: Field data for Hainich: plant area index (PAI, m^2 m^{-2}, is measured with the LAI-2000 sensor, which detects leaves and other plant organs such as fruits), leaf production (NPPl, g C m^{-2} y^{-1}), fruit production (NPPf, g C m^{-2} y^{-1}), wood production (NPPw, g C m^{-2} y^{-1}), and comments on important events in the forest (Mund M, Herbst M, Ammer C, Ghimire B, Kollascheck M, Knohl A, Schumacher J. (in preparation). It is not just a trade-off – Evidence for a complex control of vegetative and regenerative growth in an old-growth, mixed beech stand).

Year	PAI	NPPl	NPPf	NPPw	Comment
2003	6.2	175	24	199	
2004	5.5	158	100	157	masting + low spring temperatures
2005	5.8	176	8	218	
2006	6.0	163	115	190	masting
2007	5.7	179	42	254	
2008	6.5	184	10	222	
2009	6.1	175	172	194	masting
2010	6.7	181	13	175	
2011	NA	124	189	129	masting + dry spring + early bud break + late frost

Fig. S1 Time series of monthly precipitation (P; black bars) and mean monthly air temperature (Tair; grey symbols). Data were collected at the sites.

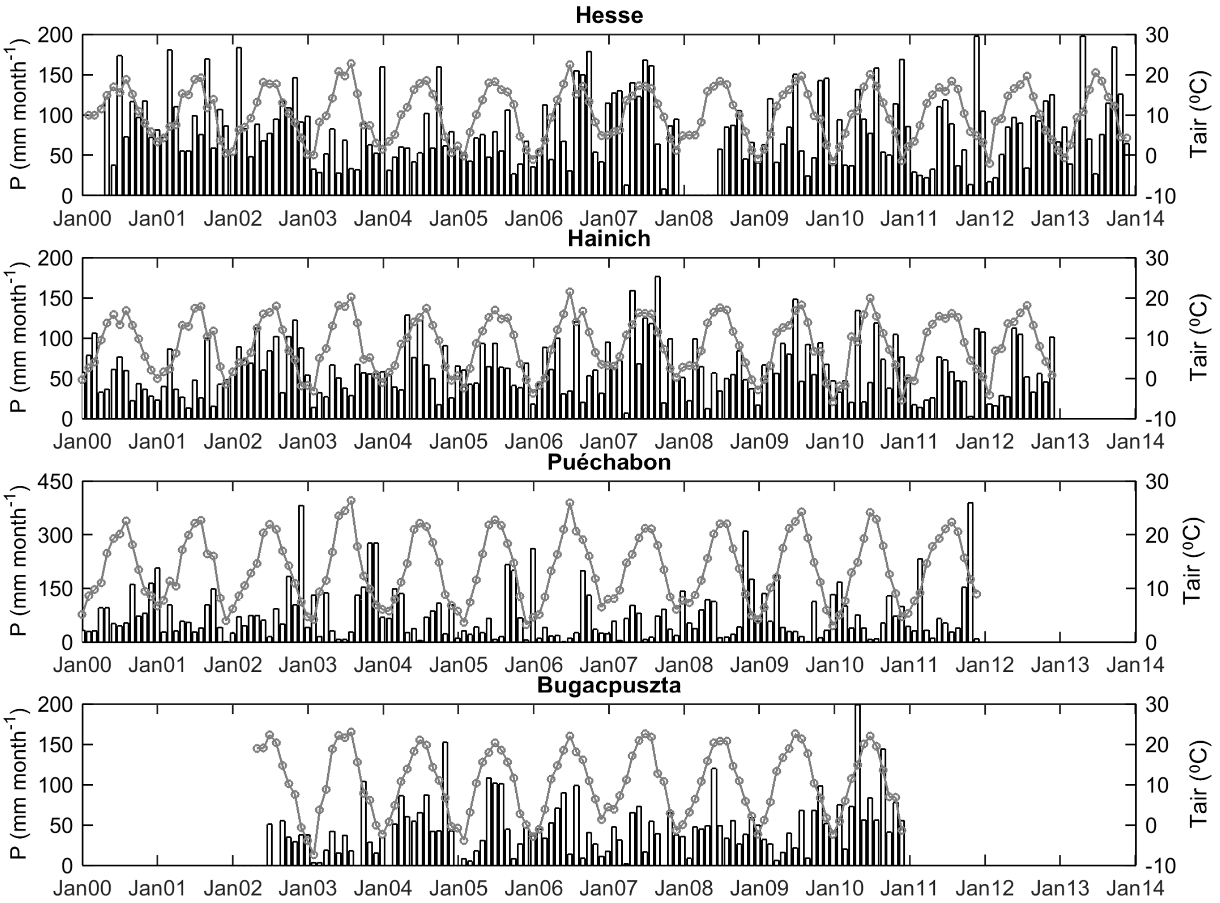

Fig. S2 Relative extractable water (REW) for Puéchabon (black line). The red line indicates the common threshold of 0.4, indicative of drought stress. See manuscript text for further details.

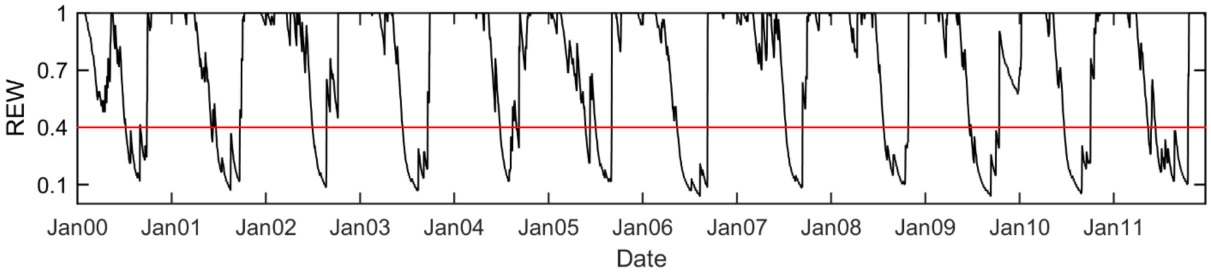

Fig. S3 Relationship between photosynthetically active radiation (PAR; µmol m^{-2} s^{-1}; obtained from flux tower) and global radiation (Rg; kJ.m^{-2}.d^{-1}; obtained from JRC-MARS), for each of the studied sites. Linear fits through the origin are shown as black lines. We used the relationship PAR=Rg/43 (red lines) to calculate PAR from the JRC-MARS data set. This method has the advantage of removing data gaps in the field data and is especially interesting for the current study where the aim is to test how drought effects observed in the field can be detected using global databases (MODIS and JRC-MARS).

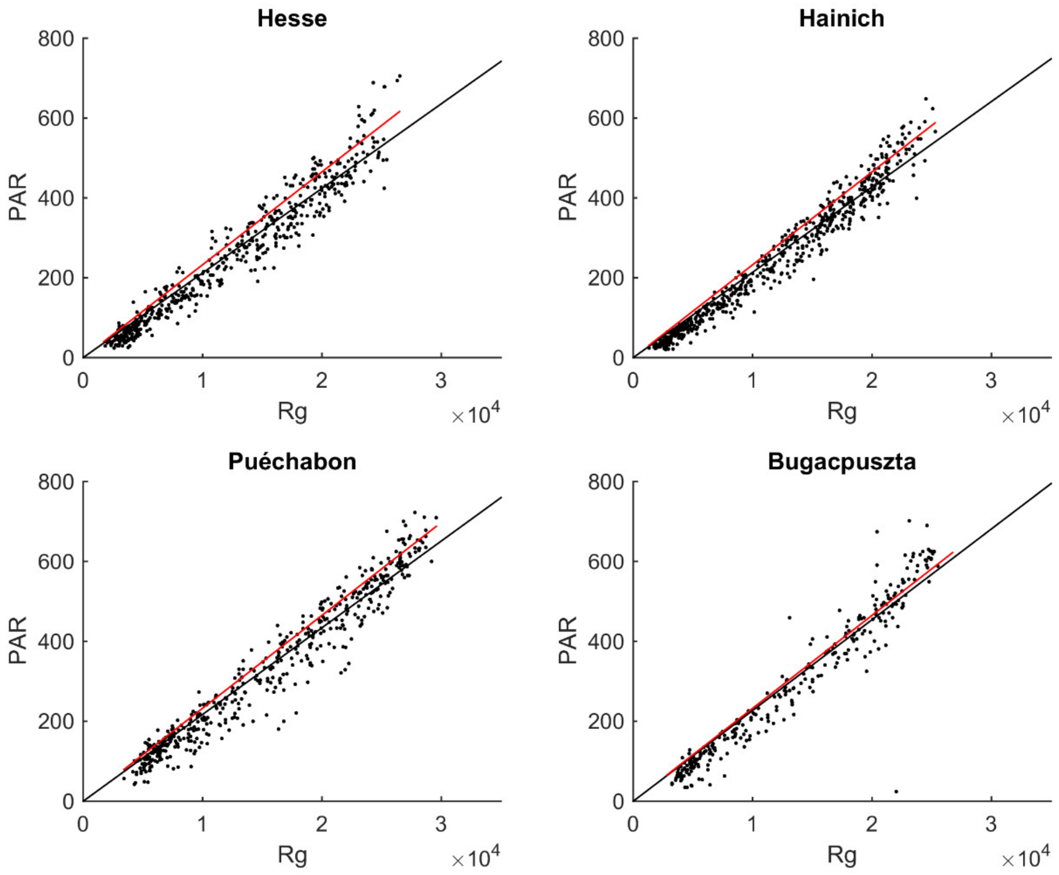

Fig. S4 Seasonal patterns The figures show the time series of flux or remotely sensed data (black points) and the average seasonal pattern (using a ~1-month moving window) used for calculating the anomalies (red lines) for the four sites.

Hesse

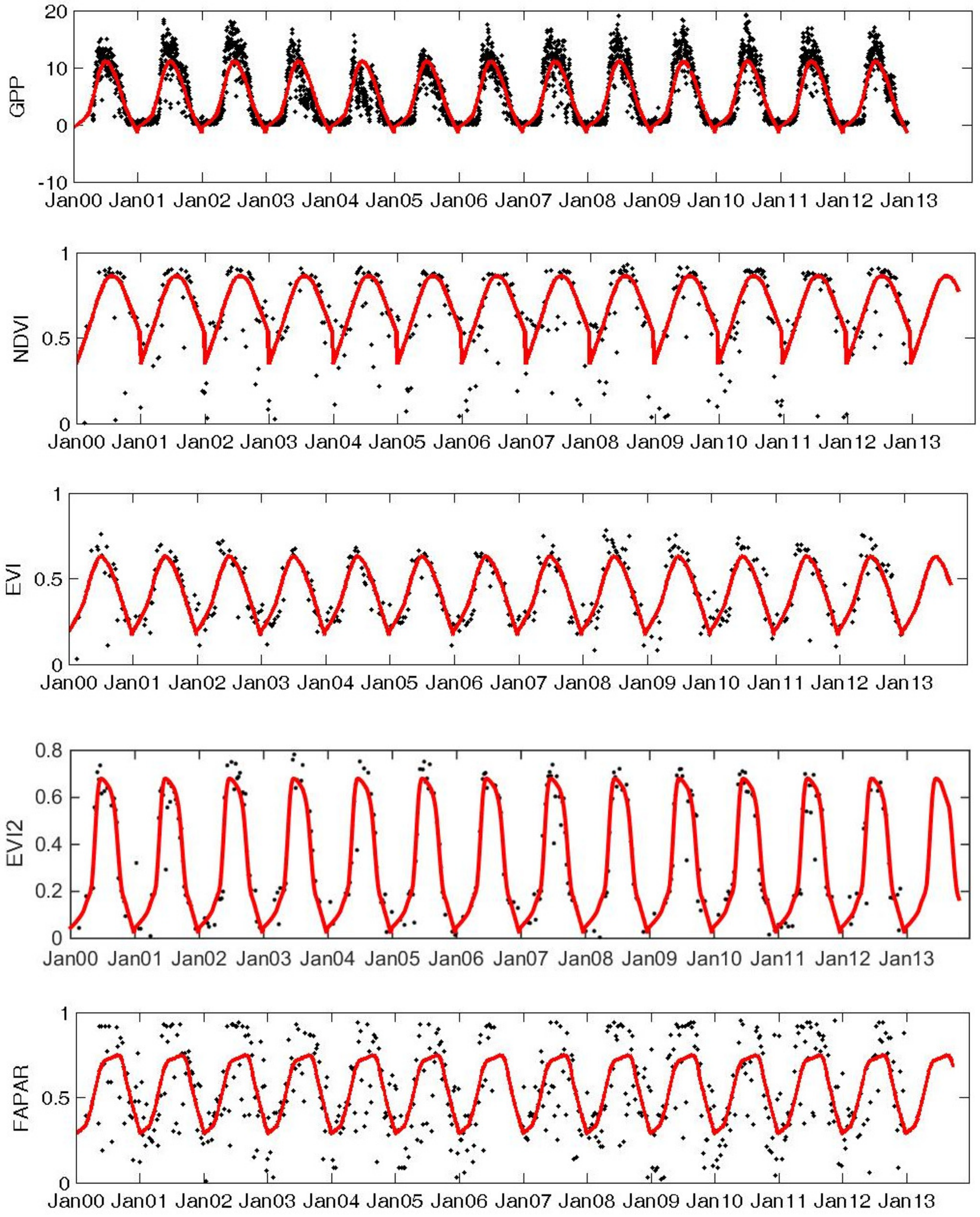

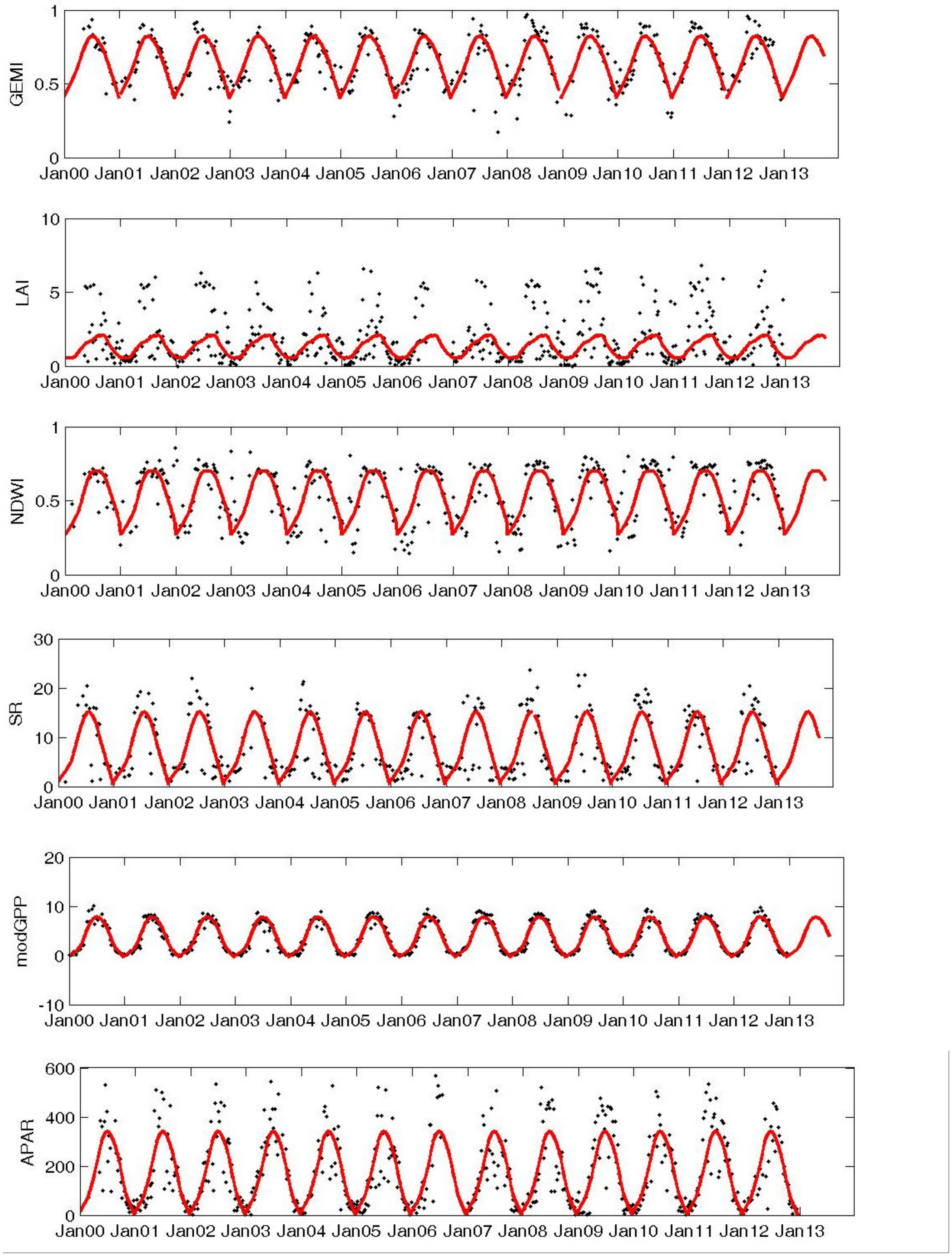
GEMI
1
0.5
0
LAI
10
5
0
NDWI
1
0.5
0
SR
30
20
10
0
modGPP
20
10
0
-10
APAR
600
400
200
0
Jan00 Jan01 Jan02 Jan03 Jan04 Jan05 Jan06 Jan07 Jan08 Jan09 Jan10 Jan11 Jan12 Jan13

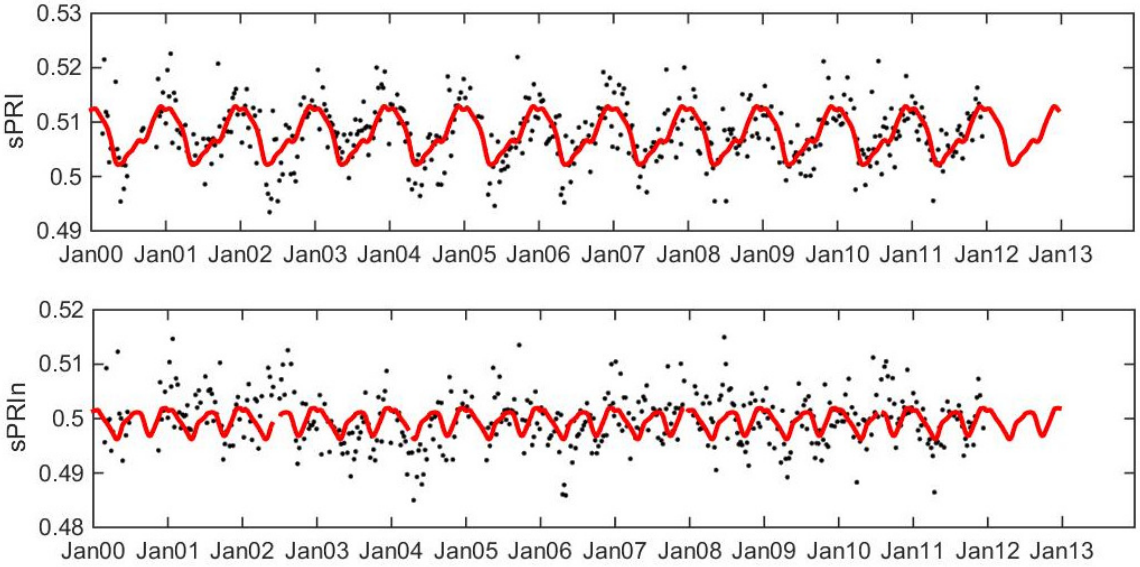
sPRI
0.53
0.52
0.51
0.5
0.49
Jan00 Jan01 Jan02 Jan03 Jan04 Jan05 Jan06 Jan07 Jan08 Jan09 Jan10 Jan11 Jan12 Jan13
sPRIn
0.52
0.51
0.5
0.49
0.48
Jan00 Jan01 Jan02 Jan03 Jan04 Jan05 Jan06 Jan07 Jan08 Jan09 Jan10 Jan11 Jan12 Jan13

Hainich

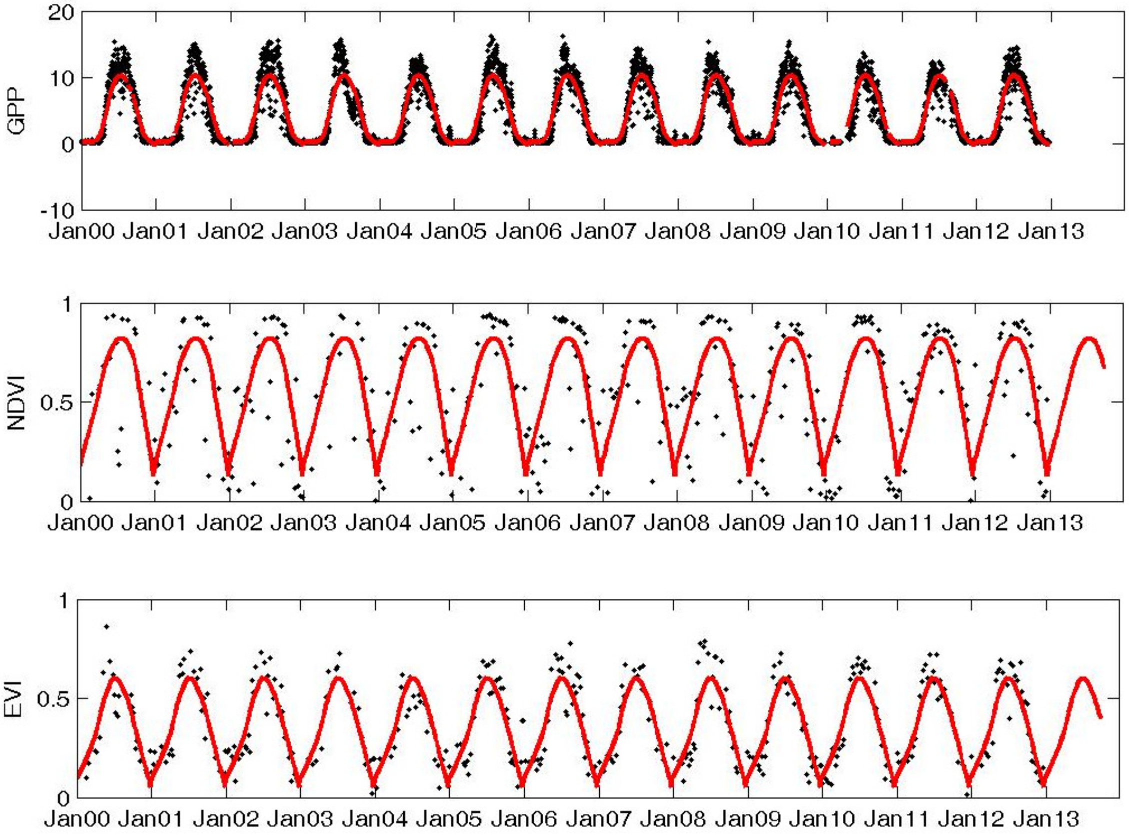
GPP
20
10
0
-10
Jan00 Jan01 Jan02 Jan03 Jan04 Jan05 Jan06 Jan07 Jan08 Jan09 Jan10 Jan11 Jan12 Jan13
NDVI
1
0.5
0
Jan00 Jan01 Jan02 Jan03 Jan04 Jan05 Jan06 Jan07 Jan08 Jan09 Jan10 Jan11 Jan12 Jan13
EVI
1
0.5
0
Jan00 Jan01 Jan02 Jan03 Jan04 Jan05 Jan06 Jan07 Jan08 Jan09 Jan10 Jan11 Jan12 Jan13

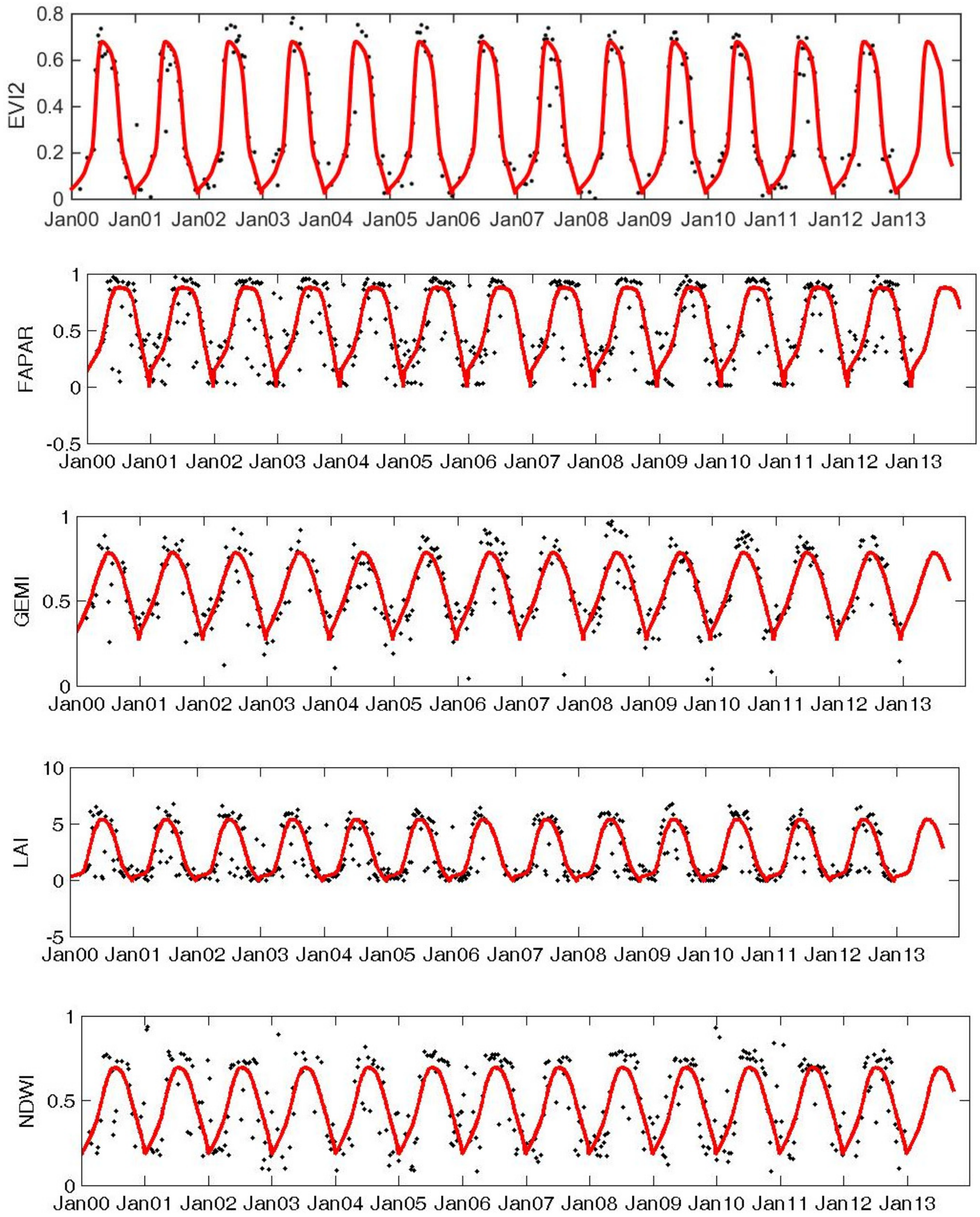
EVI2
FAPAR
GEMI
LAI
NDWI
Jan00 Jan01 Jan02 Jan03 Jan04 Jan05 Jan06 Jan07 Jan08 Jan09 Jan10 Jan11 Jan12 Jan13

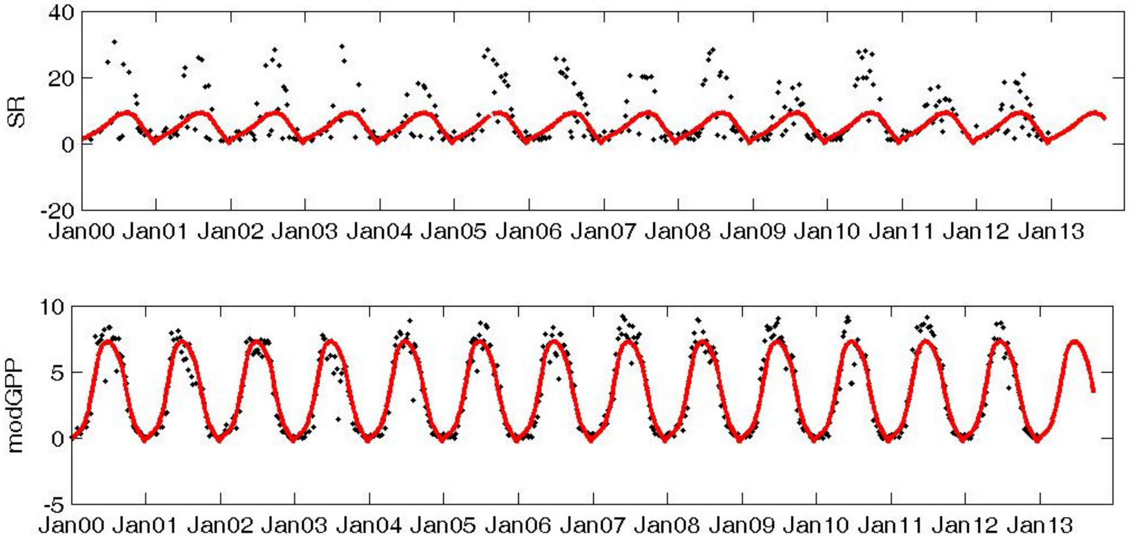
SR
40
20
0
-20
Jan00 Jan01 Jan02 Jan03 Jan04 Jan05 Jan06 Jan07 Jan08 Jan09 Jan10 Jan11 Jan12 Jan13
modGPP
10
5
0
-5
Jan00 Jan01 Jan02 Jan03 Jan04 Jan05 Jan06 Jan07 Jan08 Jan09 Jan10 Jan11 Jan12 Jan13

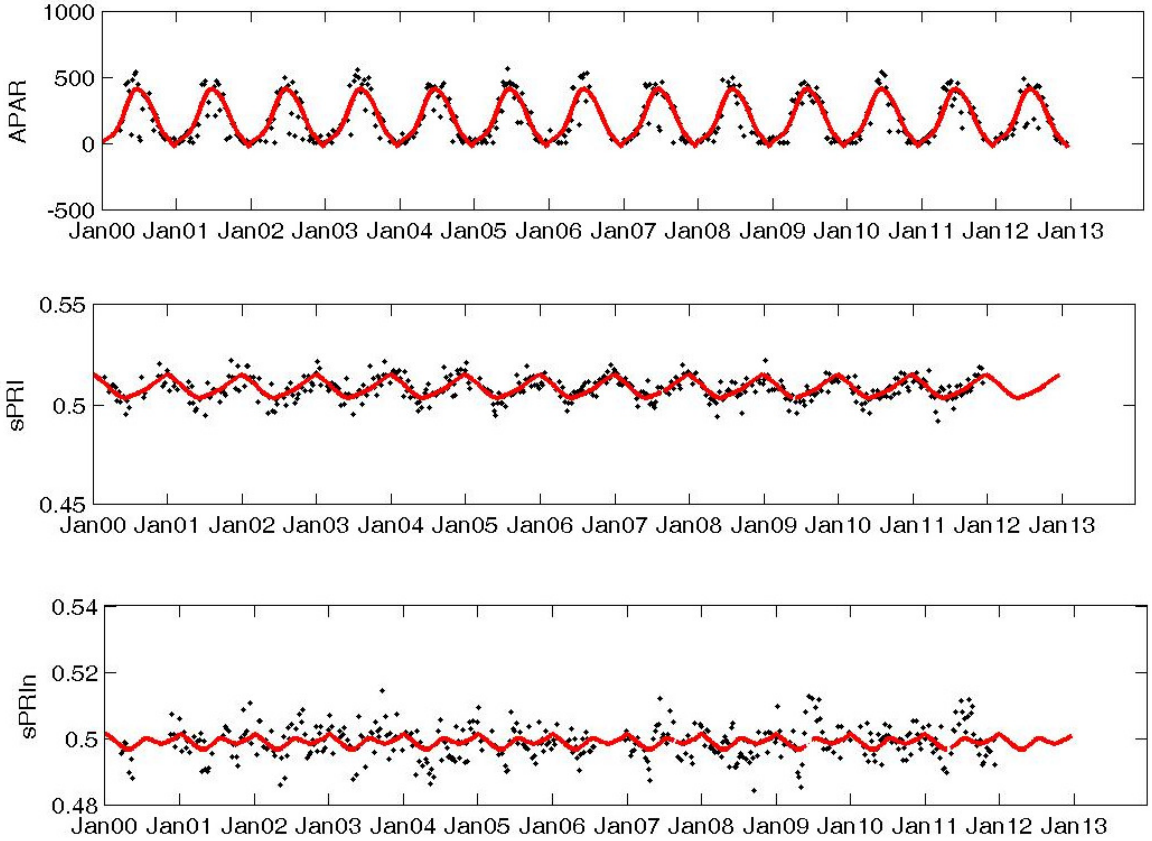
APAR
1000
500
0
-500
Jan00 Jan01 Jan02 Jan03 Jan04 Jan05 Jan06 Jan07 Jan08 Jan09 Jan10 Jan11 Jan12 Jan13
sPRI
0.55
0.5
0.45
Jan00 Jan01 Jan02 Jan03 Jan04 Jan05 Jan06 Jan07 Jan08 Jan09 Jan10 Jan11 Jan12 Jan13
sPRIn
0.54
0.52
0.5
0.48
Jan00 Jan01 Jan02 Jan03 Jan04 Jan05 Jan06 Jan07 Jan08 Jan09 Jan10 Jan11 Jan12 Jan13

Puéchabon

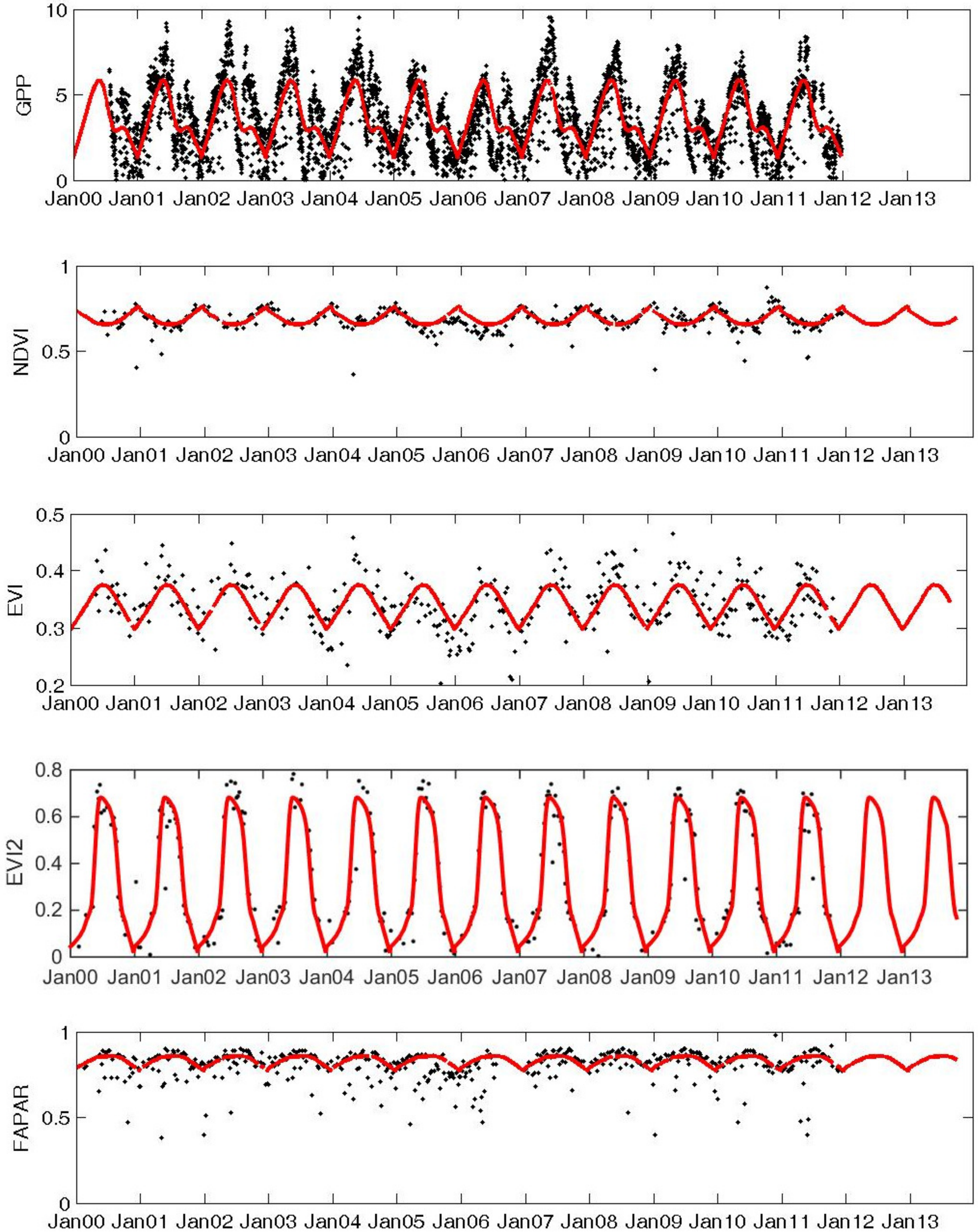
GPP
NDVI
EVI
EVI2
FAPAR
Jan00 Jan01 Jan02 Jan03 Jan04 Jan05 Jan06 Jan07 Jan08 Jan09 Jan10 Jan11 Jan12 Jan13

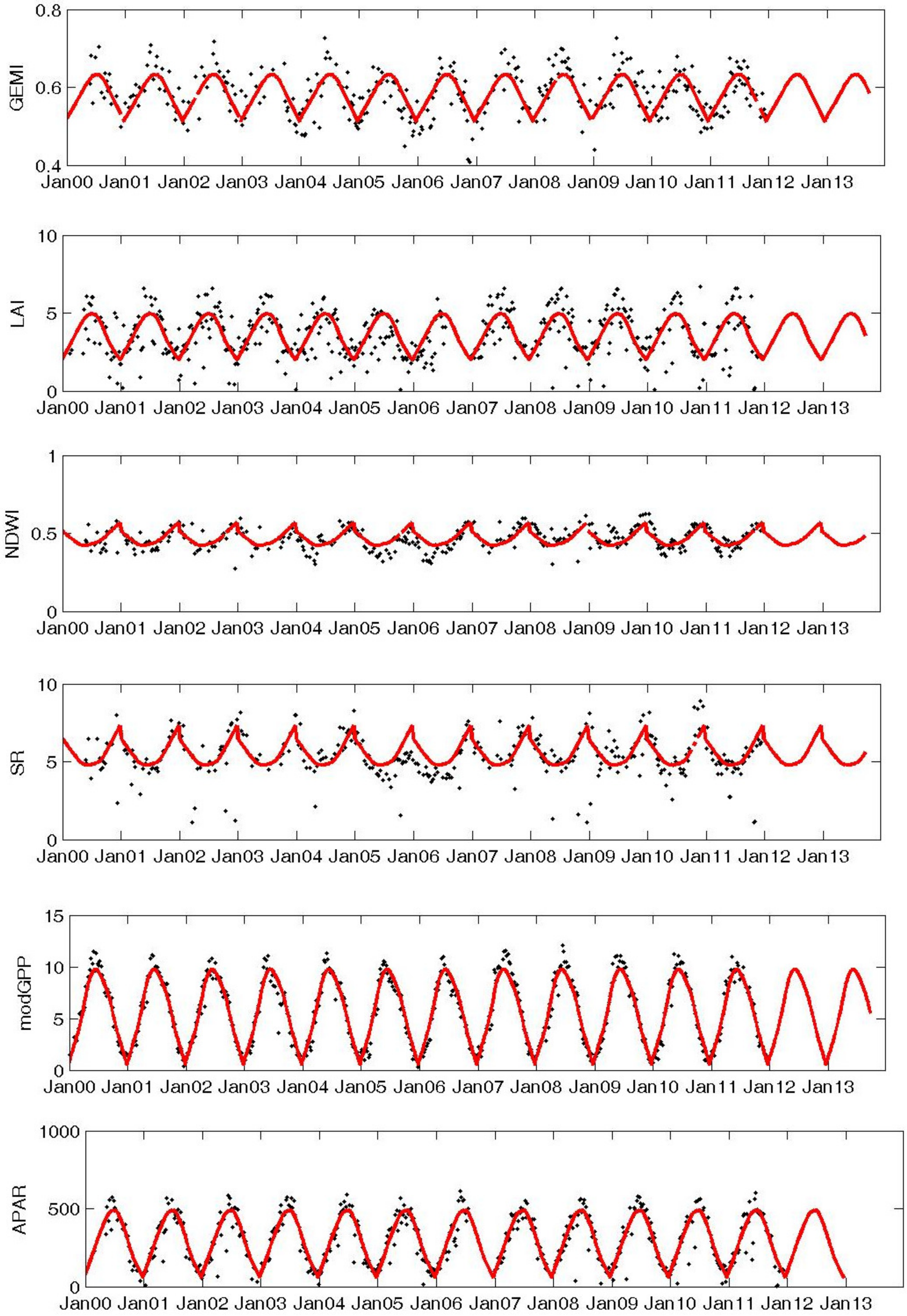
GEMI
0.8
0.6
0.4
LAI
10
5
0
NDWI
1
0.5
0
SR
10
5
0
modGPP
15
10
5
0
APAR
1000
500
0
Jan00 Jan01 Jan02 Jan03 Jan04 Jan05 Jan06 Jan07 Jan08 Jan09 Jan10 Jan11 Jan12 Jan13

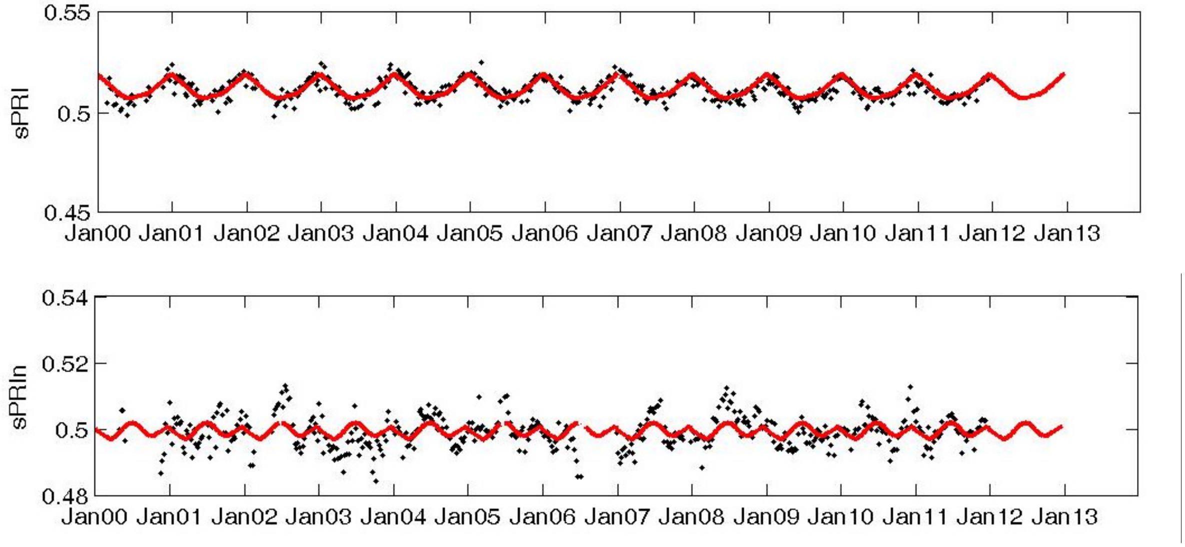

Bugacpuszta

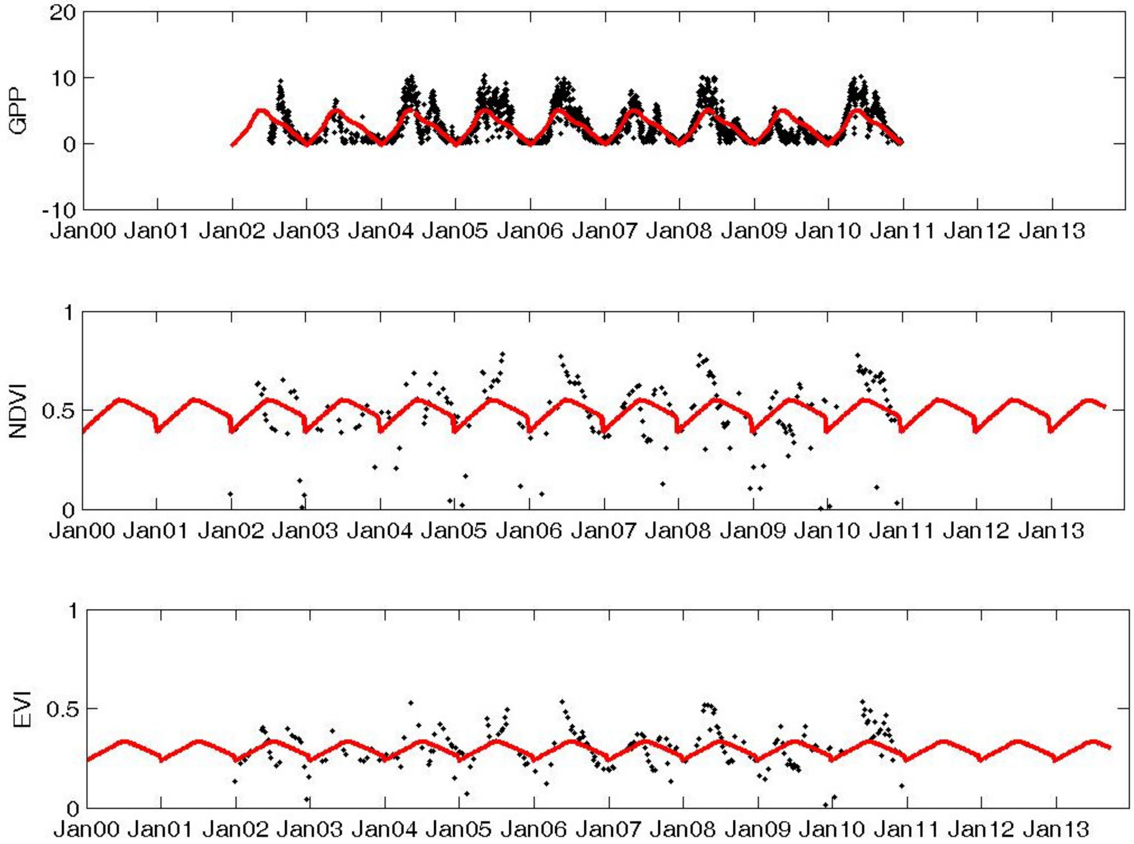

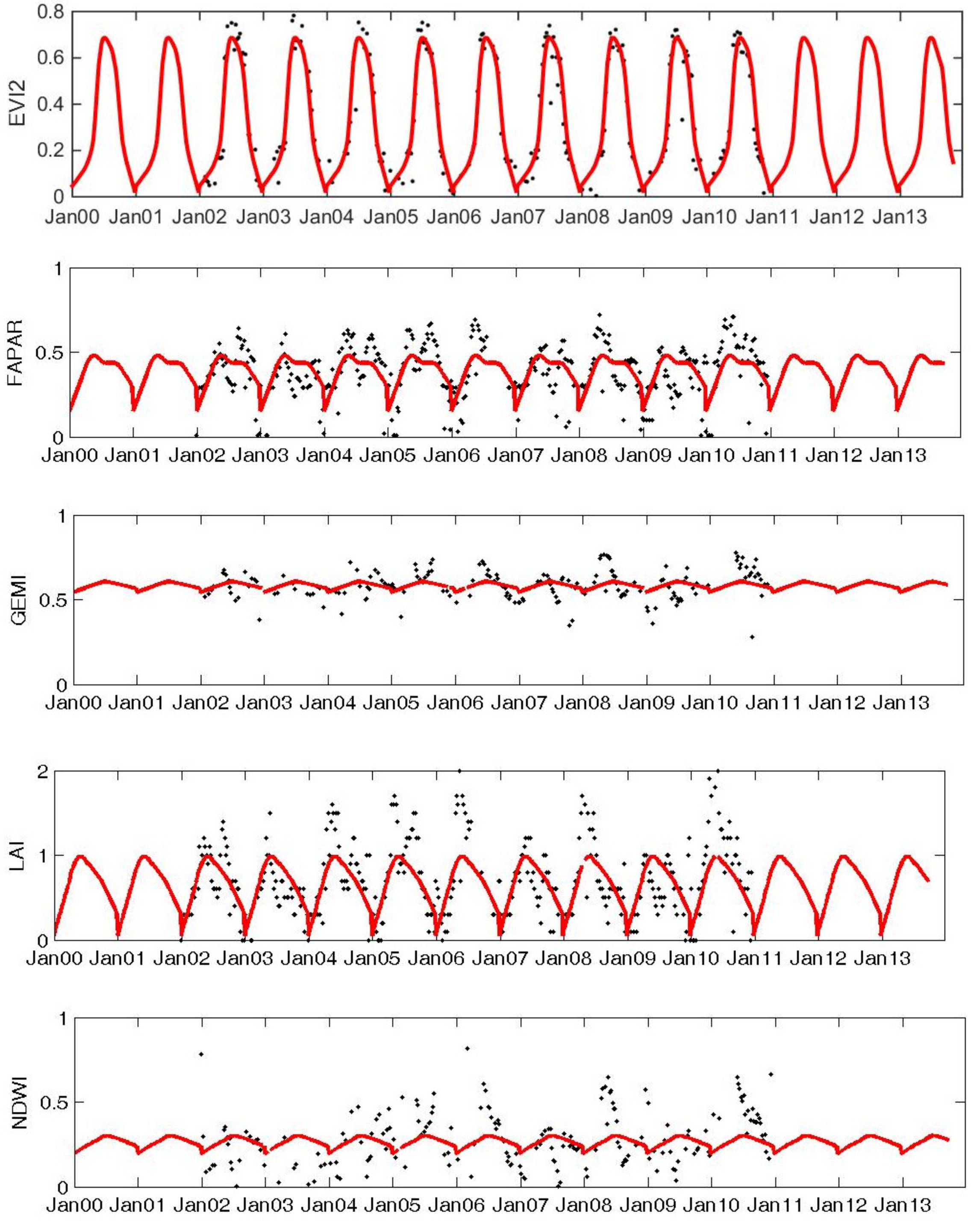
EVI2
FAPAR
GEMI
LAI
NDWI
Jan00 Jan01 Jan02 Jan03 Jan04 Jan05 Jan06 Jan07 Jan08 Jan09 Jan10 Jan11 Jan12 Jan13

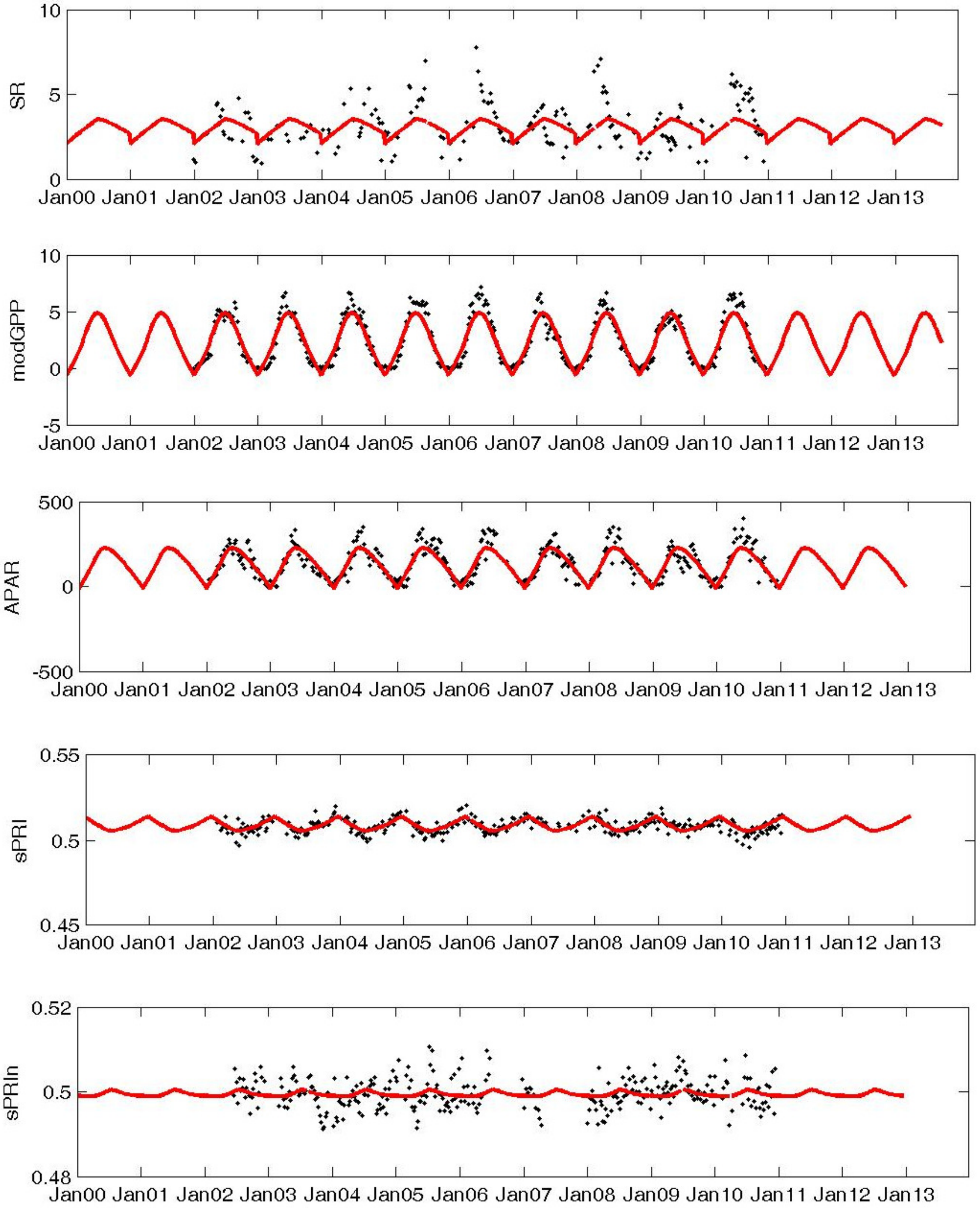
SR
modGPP
APAR
sPRI
sPRIn
Jan00 Jan01 Jan02 Jan03 Jan04 Jan05 Jan06 Jan07 Jan08 Jan09 Jan10 Jan11 Jan12 Jan13

Fig. S5 The results of the breakpoint analysis for the four sites.

Hainich

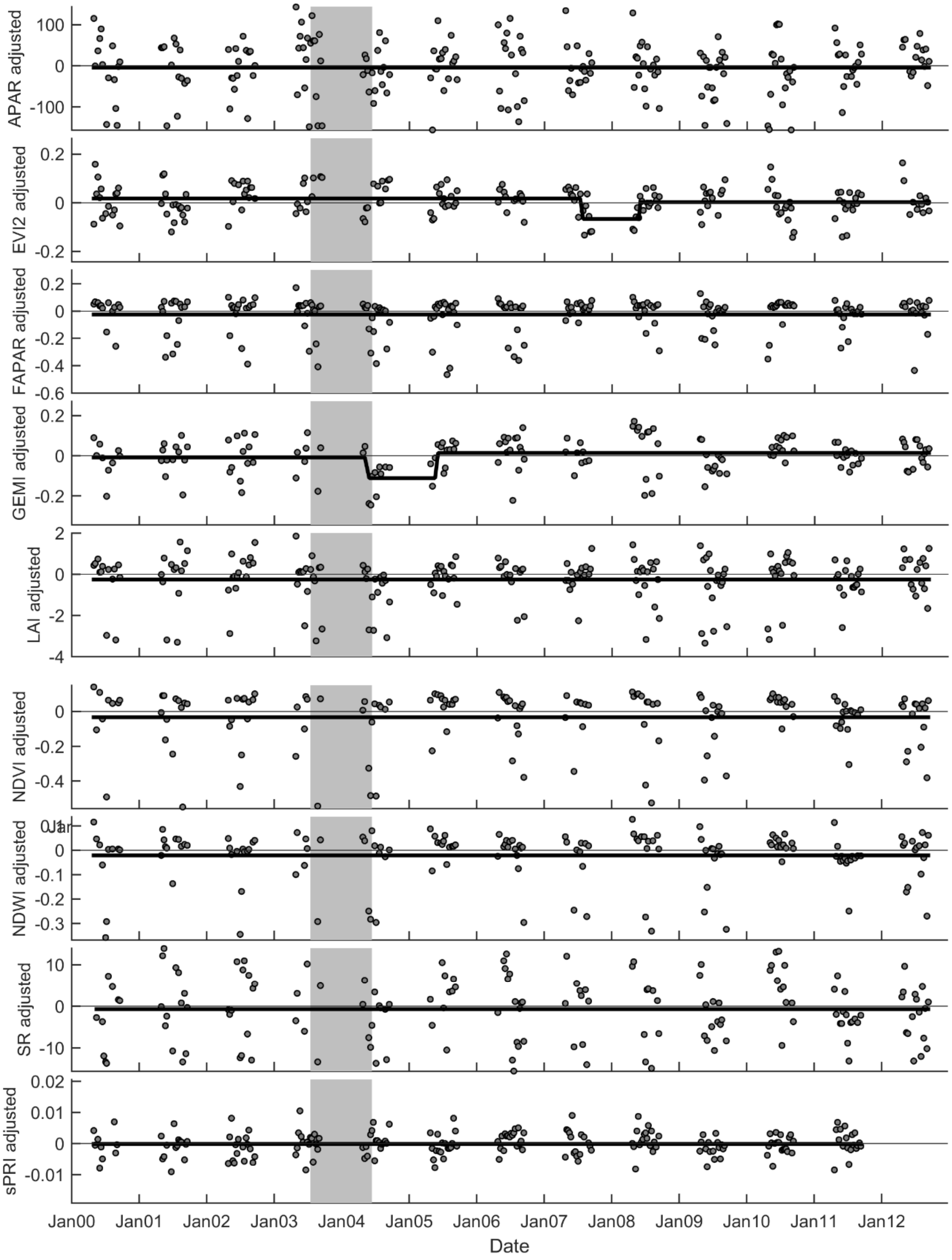

Puéchabon

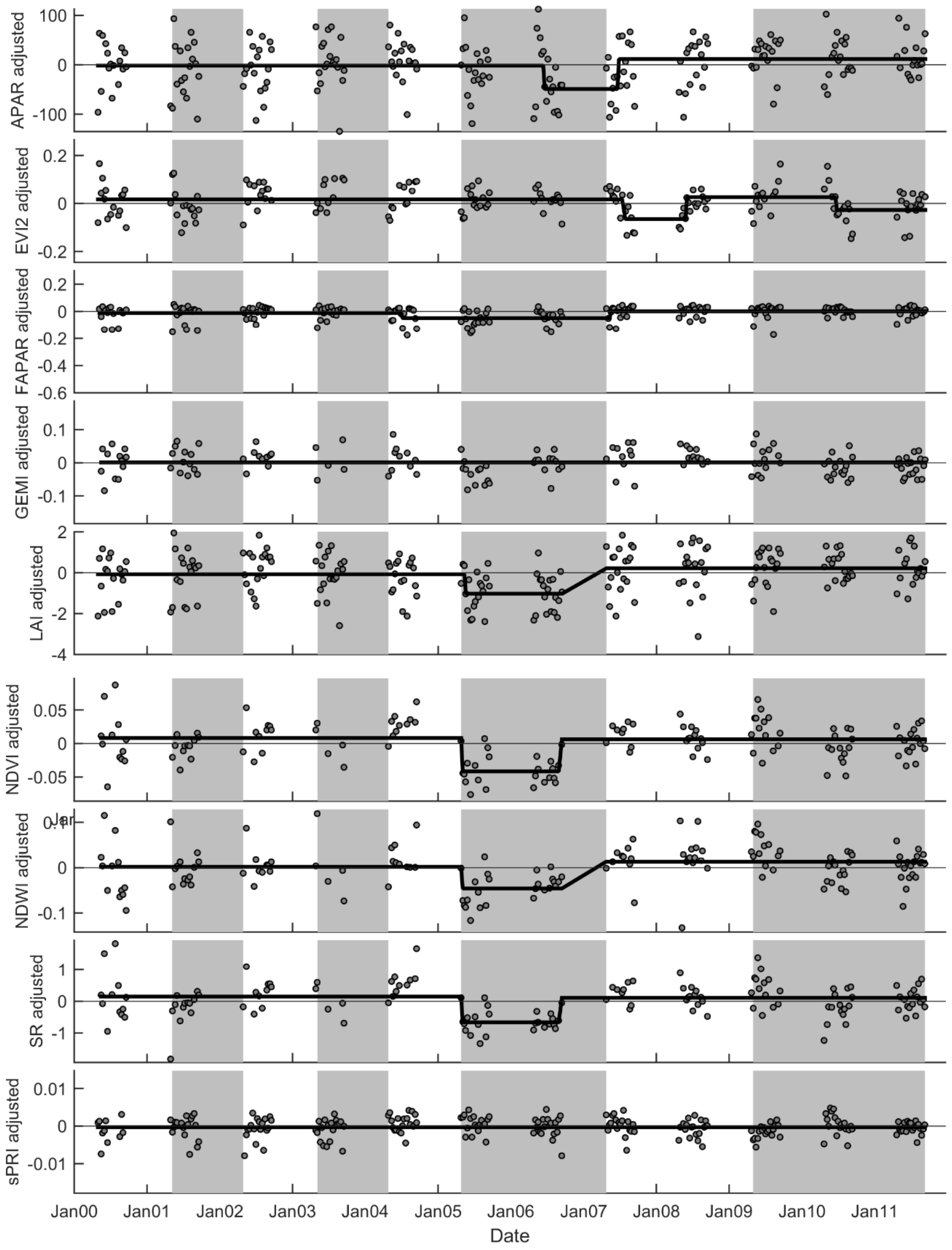
APAR adjusted
EVI2 adjusted
FAPAR adjusted
GEMI adjusted
LAI adjusted
NDVI adjusted
NDWI adjusted
SR adjusted
sPRI adjusted
Jan00
Jan01
Jan02
Jan03
Jan04
Jan05
Jan06
Jan07
Jan08
Jan09
Jan10
Jan11
Date

Bugacpuszta

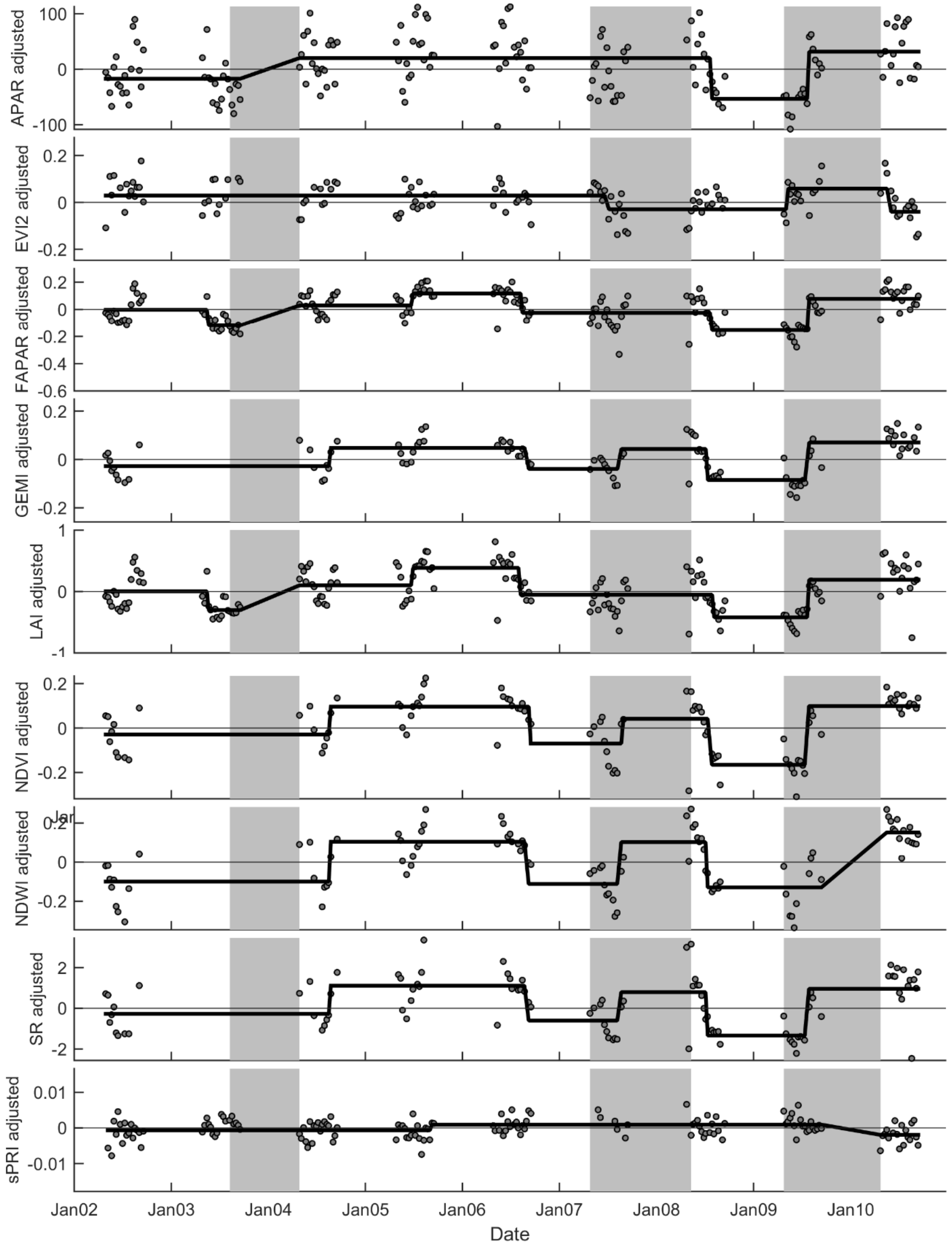

Notes S1 Water availability metrics.

Plants experience a given reduction in precipitation (or SPEI) quite differently between sites e.g., because of differences in soil texture or groundwater access; see [10], and periods of drought are therefore best identified using site-specific data. The stress experienced by plants is ideally indicated by predawn leaf water potential[10], but this measurement is not usually available. In our dataset, predawn leaf water potential was available only for Puéchabon, where they were combined with measurements of soil-water content to compute the water stress integral WSI; see [11,12]. In this forest, WSI values ranging from -100 to -150 MPa day are representative of a well-watered years, while the lowest WSI observed so far was – 358 MPa day[12]. We consider WSI ≤ -250 MPa day to be indicative of moderate to severe drought, which corresponds to a probability of 0.3 given that average WSI was -213.6 MPa day, with a standard deviation of 73.35 MPa day. Another reliable indicator of water availability is the relative extractable water (REW) in soil, which can be calculated either from soil measurements[10] or with a water balance model[13]. The latter was used for Hesse and Hainich. The necessary data to run the model were not available for Bugacpuszta, but soil-moisture measurements provided an indication of the depletion of soil moisture for part of the time series under consideration. Soil-moisture measurements are not necessarily indicative of drought as experienced by the biota (e.g., when roots explore deeper soil layers; see also[10]), but these measurements were useful for verifying the plausibility of the SPEI results.

References

1 Granier, A., Breda, N., Longdoz, B., Gross, P. & Ngao, J. Ten years of fluxes and stand growth in a young beech forest at Hesse, North-eastern France. *Ann. For. Sci.* **65**, 704 (2008).

2 Rambal, S. *et al.* Drought controls over conductance and assimilation of a Mediterranean evergreen ecosystem: scaling from leaf to canopy. *Global Change Biol.* **9**, 1813-1824 (2003).

3 Nagy, Z. *et al.* The carbon budget of semi-arid grassland in a wet and a dry year in Hungary. *Agriculture Ecosystems & Environment* **121**, 21-29 (2007).

4 Knohl, A., Schulze, E. D., Kolle, O. & Buchmann, N. Large carbon uptake by an unmanaged 250-year-old deciduous forest in Central Germany. *Agr. Forest Meteorol.* **118**, 151-167 (2003).

5 Rouse, J. W., Haas, R. H., Schell, J. A. & Deering, D. W. in *Proceedings of the Third Earth Resources Technology Satellite-1 Symposium* 3010-3017 (Greenbelt, USA; NASA SP-351, 1974).
6 Jiang, Z., Huete, A. R., Didan, K. & Miura, T. Development of a two-band enhanced vegetation index without a blue band. *Remote Sens. Environ.* **112**, 3833-3845 (2008).
7 Pinty, B. & Verstraete, M. M. GEMI - A NONLINEAR INDEX TO MONITOR GLOBAL VEGETATION FROM SATELLITES. *Vegetatio* **101**, 15-20 (1992).
8 Gao, B. C. NDWI - A normalized difference water index for remote sensing of vegetation liquid water from space. *Remote Sens. Environ.***58**, 257-266 (1996).
9 Gamon, J. A., Penuelas, J. & Field, C. B. A narrow-waveband spectral index that tracks diurnal changes in photosynthetic activity. *Remote Sens. Environ.***41**, 35-44 (1992).
10 Vicca, S. *et al.* Urgent need for a common metric to make precipitation manipulation experiments comparable. *New Phytol.***195**, 518-522 (2012).
11 Myers, B. J. Water-stress integral – a link between short-term stress and long-term growth. *Tree Physiol.* **4**, 315-323 (1988).
12 Rambal, S. *et al.* How drought severity constrains gross primary production(GPP) and its partitioning among carbon pools in a Quercus ilex coppice? *Biogeosci.***11**, 6855-6869 (2014).
13 Granier, A., Breda, N., Biron, P. & Villette, S. A lumped water balance model to evaluate duration and intensity of drought constraints in forest stands. *Ecol. Model.* **116**, 269-283 (1999).
